# Protein Evolution by Molecular Tinkering: Diversification of the Nuclear Receptor Superfamily from a Ligand-Dependent Ancestor

**DOI:** 10.1371/journal.pbio.1000497

**Published:** 2010-10-05

**Authors:** Jamie T. Bridgham, Geeta N. Eick, Claire Larroux, Kirti Deshpande, Michael J. Harms, Marie E. A. Gauthier, Eric A. Ortlund, Bernard M. Degnan, Joseph W. Thornton

**Affiliations:** 1 Howard Hughes Medical Institute, Eugene, Oregon, United States of America; 2 Center for Ecology and Evolutionary Biology, University of Oregon, Eugene, Oregon, United States of America; 3 School of Biological Sciences, University of Queensland, Brisbane, Australia; 4 Biochemistry Department, Emory University School of Medicine, Atlanta, Georgia, United States of America; University of Bath, United Kingdom

## Abstract

Understanding how protein structures and functions have diversified is a central goal in molecular evolution. Surveys of very divergent proteins from model organisms, however, are often insufficient to determine the features of ancestral proteins and to reveal the evolutionary events that yielded extant diversity. Here we combine genomic, biochemical, functional, structural, and phylogenetic analyses to reconstruct the early evolution of nuclear receptors (NRs), a diverse superfamily of transcriptional regulators that play key roles in animal development, physiology, and reproduction. By inferring the structure and functions of the ancestral NR, we show—contrary to current belief—that NRs evolved from a ligand-activated ancestral receptor that existed near the base of the Metazoa, with fatty acids as possible ancestral ligands. Evolutionary tinkering with this ancestral structure generated the extraordinary diversity of modern receptors: sensitivity to different ligands evolved because of subtle modifications of the internal cavity, and ligand-independent activation evolved repeatedly because of various mutations that stabilized the active conformation in the absence of ligand. Our findings illustrate how a mechanistic dissection of protein evolution in a phylogenetic context can reveal the deep homology that links apparently “novel” molecular functions to a common ancestral form.

## Abstract 2

Phylogenetic reconstruction of the structure and function of the ancestor of the nuclear receptor protein family reveals how functional diversity evolves by subtle tinkering with an ancestral template.

## Author Summary

Many protein families are so diverse that it is hard to determine their ancestral functions and to understand how their derived functions evolved. The existence of so many different functions within protein families often creates the impression that complex, novel functions must have evolved repeatedly and independently. Nuclear receptors (NRs) are a large family of related proteins that regulate key biological processes in animals by binding to specific DNA sequences and triggering expression of nearby target genes. Many NRs are activated by a specific hormone or other small molecule, but some do not require a ligand, and still others are incapable of activating gene expression and so act primarily as repressors of transcription. To understand how the functional diversity of NRs evolved, we reconstructed the structural and functional characteristics of the ancient protein from which the entire family evolved, using genomic, biochemical, functional, and structural analyses in a phylogenetic framework. We show, contrary to current belief, that the ancestral NR was a ligand-activated transcriptional activator that existed in the earliest period of animal evolution. Our analysis reveals how the extraordinary functional diversity of modern receptors was generated by subtle tinkering with this ancestral template—slightly reshaping the ligand cavity, stabilizing the protein's active conformation so it no longer required a ligand, or disabling the protein's capacity to activate transcription without affecting its other properties. We predict that, when sufficient data are gathered to allow detailed evolutionary reconstructions in other protein families, it will become apparent that most protein functional diversity evolved by tinkering with ancient functions; invoking the evolution of wholesale “novelty” will seldom be necessary.

## Introduction

By sequencing genomes of taxa occupying key positions in the metazoan tree of life, it has become possible to infer when important animal gene families originated and proliferated [1]–[3]. Sequence data alone, however, cannot yield insight into the functions and structures of ancient proteins or the processes by which their descendants evolved. Further, many gene families have diversified so extensively that comparisons of extant proteins from model organisms are insufficient to reveal which functions are ancestral and which are derived. In principle, it should be possible to reconstruct the history of a protein family by phylogenetically analyzing the underlying structural mechanisms that produce functional diversity among densely sampled members of the family. Such a strategy would be analogous to detailed studies of the evolution of animal development, which have revealed the deep homology of diverse morphologies in distant lineages and the mechanisms by which they evolved from common ancestral forms [4].

The members of the superfamily of nuclear receptor (NR) transcription factors, for example, are regulated in diverse ways—by ligands, postranslational modifications, and association with other proteins or DNA—depending on the cellular context [5]. NRs have a modular domain structure, including a highly conserved DNA-binding domain (DBD) and a moderately conserved ligand-binding domain (LBD)—which in most receptors contains a ligand-regulated transcriptional activation function—along with extremely variable hinge and N-terminal domains. There is considerable diversity in the functions of NR LBDs, which can be roughly classified into three major groups. In one class, the LBD's transcriptional function can be activated by a specific hydrophobic ligand, such as a steroid, retinoid, or fatty acid; the ligand binds in a deep internal cavity, remodeling and stabilizing the LBD's conformation to generate a new binding surface for coactivator proteins, which increase transcription of nearby genes [5]. The second class of NRs are ligand-independent transcription factors, often called “constitutive” receptors, the LBDs of which can adopt the active conformation and activate gene expression in the absence of a ligand or other modifications. Some members of this class lack the internal cavity and are not known to bind any ligands, whereas others do bind hydrophobic molecules, which up- or down-regulate their baseline activity [6]–[9]. In the third class of NRs, the LBD lacks the capacity to interact with coactivators, so these receptors function primarily as transcriptional repressors that occupy NR response elements or dimerize with and thereby silence other NRs [10]–[12].

It is widely believed that the NR superfamily evolved from a ligand-independent transcriptional activator, with binding of different ligands gained independently in numerous NR lineages [13],[14]. The alternate view—that NRs evolved from a liganded ancestor, with ligand-dependence lost in the lineages leading to the ligand-independent receptors—has received little attention. These two hypotheses exemplify opposite views on the general nature of molecular evolution and the origin of complex functions. The hypothesis that the ancestral NR was ligand-independent implies that a complex molecular function—allosteric regulation of transcription by binding a ligand—evolved de novo many independent times, requiring evolution to repeatedly create novelty and complexity [15],[16]. In contrast, the hypothesis of a ligand-activated ancestor implies that evolution produced new functions primarily by subtle tinkering with a conserved ancestral mechanism [4],[17], which allowed receptors to accommodate new molecular partners or lose dependence on those partners because of mutations that modified or degraded existing functions.

Several limitations have impeded rigorous inference about the ancestral NR's characteristics and the diversification of the superfamily. First, the root of the gene family phylogeny has been ambiguous, leaving unknown the location of the ancestor relative to its descendants. Second, the topology of the NR phylogeny has been uncertain, because of limited sequence sampling and/or use of outdated phylogenetic methods. Third, the functions of NRs in taxa branching near the root of the metazoan phylogeny have not been characterized. Finally, whether distantly related NRs with similar functions share homologous or convergent underlying mechanisms has not been determined. Recently acquired information—including genome sequences from basal metazoans and extensive data on NR structures and functions—along with improved algorithms for phylogenetic analysis of large datasets, now allow these barriers to be overcome. Here we report on biochemical, functional, structural, and phylogenetic analyses of the NR superfamily, which allow us to reconstruct the characteristics of the ancestral nuclear receptor and understand how the functional diversity of NR LBDs evolved.

## Results/Discussion

### NRs in the Sponge Genome

The root of the NR phylogeny has been unknown because of uncertainty about the relative ages of the various NR family members. NRs appear to be a metazoan innovation, because they are absent from the genomes of choanoflagellates, fungi, plants, and prokaryotes. Until recently, however, all fully sequenced animal genomes have come from protostomes and deuterostomes, both of which contain virtually all the major NR subfamilies [18]; these data indicate only that most NR diversity was already established by the time of the protostome-deuterostome ancestor. To determine the most ancient NR lineages, we identified NRs in the newly sequenced genome of the demosponge *Amphimedon queenslandica—*a representative of the Porifera, the most anciently branching metazoan phylum based on whole-genome phylogenies [19]. We found that the *A*. *queenslandica* genome contains two NRs, which we refer to as AqNR1 and AqNR2. We amplified transcripts of each by polymerase chain reaction, verified their sequences, and analyzed their developmental expression using in situ hybridization. AqNR2 is expressed ubiquitously, whereas AqNR1 is expressed in a range of cells that contact the external environment and possess apico-basal polarity (Figure S1). We also identified NRs in genomes from two other recently sequenced early-branching lineages, the placozoan *Trichoplax adhaerens* and the cnidarian *Nematostella vectensis*, which contain 4 and 17 NRs, respectively (see also [20]). These results point to very limited NR diversification before the origin of the Eumetazoa and indicate that basal metazoan species have the potential to shed light on early NR evolution.

### Phylogeny of the NR Superfamily

**Figure 1:**
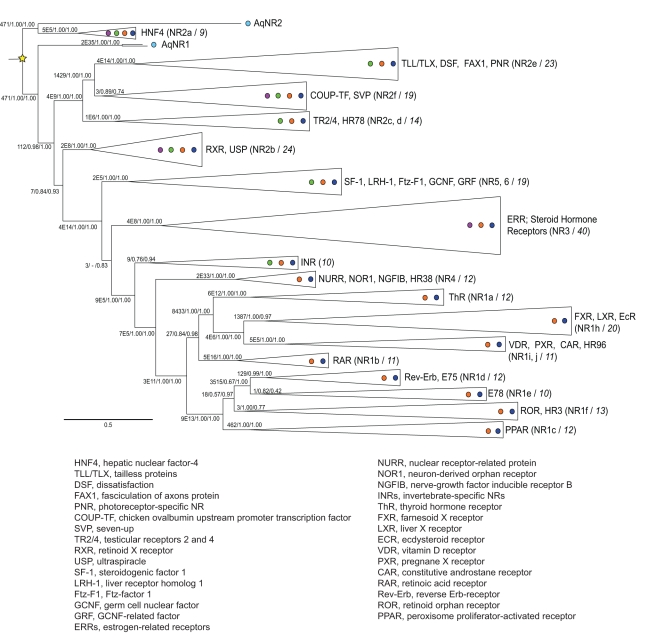
Phylogeny of the NR superfamily. A reduced representation of the NR phylogeny inferred by maximum likelihood and Bayesian method is shown. Colored circles indicate the presence of each clade in major metazoan taxa: sponges (light blue), placozoans (purple), Cnidaria (green), protostomes (orange), and deuterostomes (dark blue). Clades are labeled with their common protein names; NR nomenclature codes are in parentheses (see also Table S6), with the number of sequences analyzed in each group after the slash. Branch labels show support measured as approximate likelihood ratios (the ratio of the likelihood of the best tree with that node to the best tree without it), Bayesian posterior probabilities, and chi-square confidence estimates (the probability of a likelihood ratio at least as great as the observed ratio if the node is not resolved on the true tree). INRs, clade of invertebrate-only nuclear receptors with no standard nomenclature. Scale bar, probability of substitutions per site. A key to abbreviations of protein names is shown below the phylogeny. For unreduced phylogenies and a list of species, genes, accessions, and receptor abbreviations, see Figures S2–S3 and Tables S1 and S6.

To determine the phylogeny of the NR superfamily, we used model-based phylogenetics to analyze a taxonomically diverse database of 275 NR protein sequences (Figure 1, Table S1). The alignment includes the DBDs and LBDs of the complete NR complements in 11 sequenced genomes from eight broadly sampled animal phyla, plus NRs from 30 other species strategically chosen to maximize phylogenetic accuracy and minimize redundant signal [21]. Unlike previous studies, which used sparser sequence sampling and/or less powerful methods [14],[18],[22], phylogenetic analysis of this alignment using maximum likelihood yielded a well-resolved phylogeny (Figures 1, S2) with strong support for the placement of the basal metazoan sequences and for the relationships among most major NR families. (A few aspects of the topology, however, had weak support, such as whether the SF-1 class has a monophyletic or paraphyletic relationship to the group containing the steroid hormone receptors.) We also conducted Bayesian Markov Chain Monte Carlo (BMCMC) methods using a slightly smaller 174-sequence dataset, assembled by removing sequences at the ends of very long branches and multiple orthologs within the same phylum. This analysis recovered a nearly-identical phylogeny to the maximum likelihood analysis (Figures 1, S3).

The phylogeny is unlikely to be an artifact of the presence of rapidly evolving sites or taxa. When the 40% of sites with the fastest evolutionary rates were removed from the analysis, only the placement of the RXR group was affected (Figure S4). Further, maximum likelihood analysis of the reduced 174-sequence dataset—from which the longest terminal branches were removed—yielded the same phylogenetic relationships as the unreduced analysis (Figure S5).

### Expansion of NRs Early in the Metazoa

**Figure 2:**
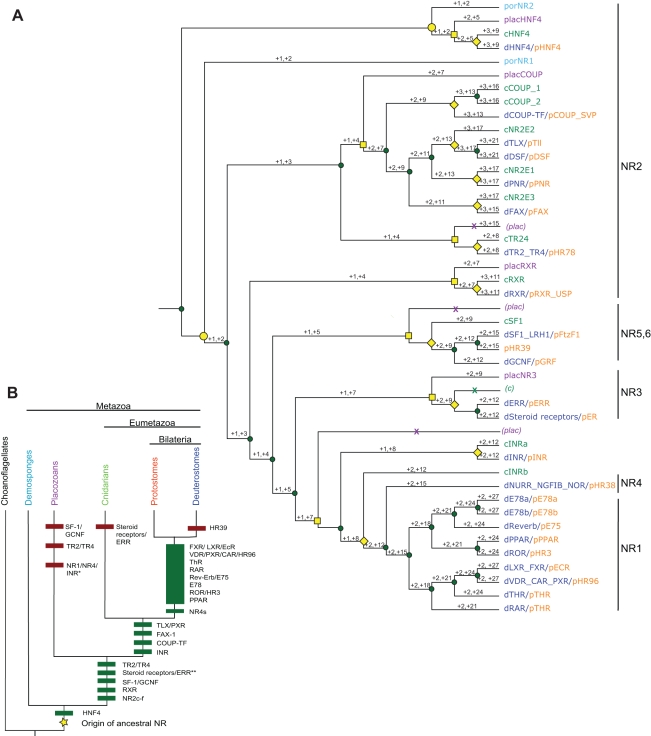
Duplications and losses in NR evolution. (A) Reconciliation of NR gene family phylogeny with the species tree shown in panel B. The phylogeny is rooted to minimize the total number of gene duplications and losses; each branch is labeled with the number of additional duplications and losses required for the tree to be rooted on that branch. Gene duplications are shown as green circles, gene losses as Xs, and speciation events as yellow shapes (circles, split of demosponges from eumetazoans; squares, split of placozoans from eumetazoans (*s.s.*); diamonds, split of cnidarians from bilaterians). Colors of gene names indicate the taxon from which they are derived, using the color scheme in panel B. (B) Gene duplication history implied by the reconciled tree in panel A. Green bars, duplications; red bars, losses. Duplications are labeled with the named NR lineages they generated. The NR1/NR4/INR ortholog lost in the placozoans (marked *) was generated in the duplication marked **. The large bar comprises numerous duplications that cannot be temporally ordered.

The phylogeny can be rooted at a single most parsimonious location between AqNR1 and AqNR2, allowing the ancestral NR to be located on a specific branch of the phylogeny. This rooting provides a coherent history of NR expansion by gene duplication with few subsequent losses (Figure 2). All alternative rootings that place other NR lineages in a basal position require many additional duplications and losses (Figure 2A). For example, placing the clade of ligand-independent estrogen related receptors (ERRs) as the outgroup requires two additional duplications and 12 additional losses compared to the optimal root; placing the ligand-independent NR4 class as the outgroup requires two additional duplications and 15 additional losses.

The phylogeny indicates that AqNR2 is orthologous to the fatty acid-binding HNF4 family and that AqNR1 is the unduplicated ortholog of all other NRs. This phylogeny indicates that the last common ancestor of all Metazoa contained two NRs—one ortholog of HNF4 and one of AqNR1, which subsequently gave rise to all other NR classes (Figure 2). After the divergence of demosponges from other metazoans but before the split of Cnidaria from the Bilateria, nine more duplications gave rise to most of the major recognized NR types, except for those in the named classes NR1 and NR4, which proliferated during the interval between the cnidarian-bilaterian ancestor and the protostome-deuterostome ancestor (Figure 2). Many NR subfamilies diversified further within the vertebrates.

Support for the placement of AqNR2 with the HNF4s and of AqNR1 as sister to all other NRs is strong, with posterior probabilities of 1.0 and 0.98, chi-square confidence values of 1.0 and 1.0, and approximate likelihood ratios of 471 and 112, respectively (Figures 1, S2–S3). The next-best rearrangement of the relationships between the sponge receptors and the rest of the NRs has a likelihood several orders of magnitude lower than that of the ML tree. This alternate phylogeny (Figure S6) would place AqNR1 and AqNR2 as sister paralogs specific to the sponge lineage. It would imply that the ancestral metazoan contained a single NR; duplication of this ancestral gene in the sponges would have yielded AqNR1 and AqNR2, and the first duplication that separated the HNF4 group from the rest of the NR superfamily would have occurred in the Eumetazoa after they diverged from demosponges. The rest of the superfamily's history would remain unchanged.

### Functional Characterization of Demosponge NRs

**Figure 3:**
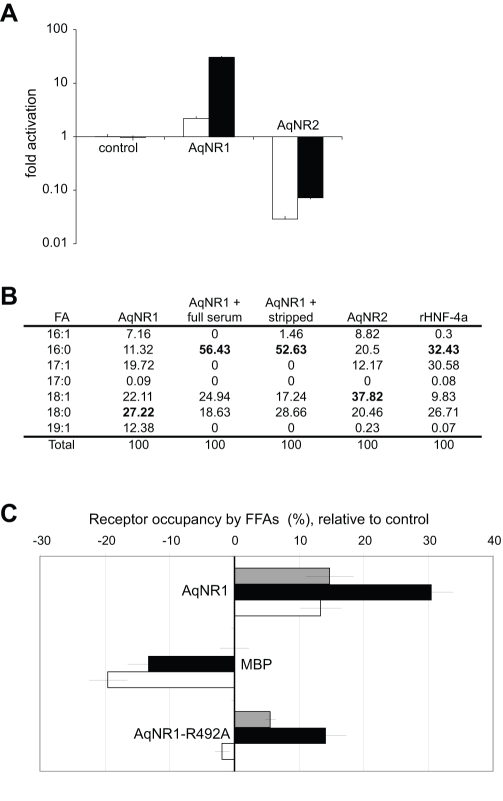
Ligand binding and transcriptional activation by *A. queenslandica* nuclear receptors. (A) Transcriptional effects of AqNR1 and AqNR2 LBDs in a luciferase reporter assay. Transfected cells were treated with stripped or complete serum (white and black bars, respectively). Activation is scaled relative to vector-only control; error bars, SEM for 3 replicates. (B) Fatty acid (FA) binding profile by AqNR1 and AqNR2, as determined by electrospray ionization/mass spectrometry. Binding of each FA species is shown as a percentage of the total FAs bound by each receptor. The most abundantly bound FA for each receptor is in bold. FA binding by rat HNF4α is shown for comparison. Measurements are shown relative to a labeled palmitic acid standard and represent the average of two runs. (C) Quantitative fatty acid binding by AqNR1 and mutant AqNR1-R492A as determined in a colorimetric enzymatic assay. Receptors were expressed in *E. coli*, purified, and treated with no serum (gray), complete serum (black), or stripped serum (white). Errors bars, SEM for 3 replicates. The percentage of receptor molecules occupied by FAs (after subtraction of background binding by MBP/no serum control) is shown for each experimental condition.

Hypotheses concerning the functions of the ancestral proteins are strongly affected by the functions of proteins that branch off the family phylogeny near its root. To understand how the functions of the NR LBD evolved, we experimentally characterized the capacity of AqNR1 and AqNR2 LBDs to bind and be regulated by ligands. In a reporter gene assay, AqNR1 had very weak intrinsic activity—less than 2-fold activation—when incubated with serum from which hydrophobic small molecules were stripped using dextran-charcoal. When treated with complete serum, however, AqNR1 transcription increased by 30-fold, suggesting that the receptor is activated by a hydrophobic ligand that is present in mammalian serum, such as a fatty acid or steroid (Figure 3A).

To characterize AqNR1's potential ligand, we expressed and purified AqNR1-LBD in bacteria, extracted bound hydrophobic molecules, and used mass spectrometry to identify the bound compounds. Like mammalian HNF4 [23], AqNR1-LBD bound an array of bacterial free fatty acids (FAs) with tail lengths ranging from 16 to 19 carbons, with preference for 18∶0 and 18∶1 fatty acids (Figures 3B, S7). When AqNR1 was incubated with complete mammalian serum, palmitic acid was the dominant FA bound, along with lower proportions of 18∶0 and 18∶1 FAs (Figure 3B). We then confirmed and quantified FA binding by the purified AqNR1-LBD using an enzymatic assay. As predicted, we found that AqNR1-LBD binds both *E. coli* and mammalian FAs; receptor occupancy by FAs approximately doubles when the protein is treated with complete serum but does not increase upon treatment with stripped serum (Figure 3C).

**Figure 4:**
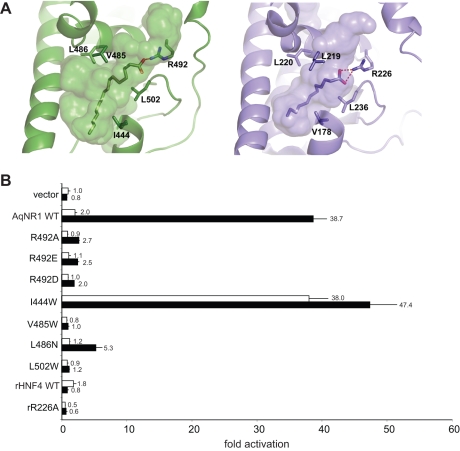
Conserved architecture and function of the AqNR1 ligand pocket. (A) The ligand cavity in the predicted structure of AqNR1 (green, with palmitic acid) and in the crystallographic structure of rat HNF4α (blue, with lauric acid) are shown. Cavities (volumes of 835 and 450 Å^3^ , respectively) are shown as surfaces, and the ligands as sticks. Labeled side chains at homologous positions that contact the ligand were subject to experimental characterization. Dashed lines show hydrogen bonds. (B) Effect of mutagenesis of ligand-contacting residues on transactivation in a luciferase reporter assay by AqNR1 and rat HNF4α in stripped (white bars) and complete serum (black bars). Bar labels show fold activation relative to vector-only control with stripped serum; error bars, SEM for 3 replicates.

To more directly test the hypothesis that AqNR1 binds and is activated by FAs, we characterized the functional effects of mutations in the predicted AqNR1 ligand pocket. We first predicted the structure of AqNR1-LBD using a homology model based on the X-ray crystal structure of mammalian HNF4, the NR with the highest sequence similarity to AqNR1. The predicted structure (Figure 4A) indicates that AqNR1 is likely to have a large ligand pocket (835 Å^3^) with ample space to accommodate FAs. As in the crystal structure of mammalian HNF4s, the FA in AqNR1 is predicted to be coordinated by a hydrogen bond from a conserved arginine (Arg226 in rat HNF4α, Arg492 in AqNR1) to the FA's carboxylate oxygen; further, packing interactions between hydrophobic amino acids that line the pocket and the ligand's tail are also conserved.

We then used directed mutagenesis and functional assays to experimentally test the hypothesis that AqNR1 binds and is activated by FAs in a manner conserved with mammalian HNF4. As predicted, when the basic Arg492 was mutated to alanine, reporter activation by AqNR1 in the presence of complete serum was abolished (Figure 4B), and FA binding by the purified protein was dramatically reduced (Figure 3C). Replacement of Arg492 by several other amino acids, each of which would remove the hydrogen bond to the FA's carboxyl oxygen, also abolished reporter activation by AqNR1 (Figure 4B). Although rat HNF4α is a weaker activator in this cell line than AqNR1, mutations at this site in HNF4α also reduced activation, consistent with a common structural mode of ligand-binding (Figure 4B). Rat HNF4α, which is thought to be activated by fatty acids produced endogenously in liver cells [24], is not further activated by complete serum, indicating that its specific ligand is different from that of AqNR1 (Figure 4B).

We also mutated several hydrophobic residues in AqNR1 that are predicted to contact the FA's tail; as expected, activation by complete serum was dramatically reduced (Figure 4B). One bulky mutation in the predicted pocket, I444W, conferred strong activity on AqNR1 in the presence of stripped or full serum, implying that this mutation stabilizes the active conformation without ligand or allows binding of an unknown ligand that is present in the cultured cells or medium. Taken together, these data indicate that AqNR1's transcriptional activity is affected by binding of a hydrophobic ligand, that the ligand may be a FA, and that key aspects of the AqNR1's structure-function relations are largely conserved with those of mammalian HNF4. Identification of the specific natural ligand for AqNR1—like that of the ligand for mammalian HNF4 [25]—requires further research, as does determination of whether that ligand has an endogenous or exogenous source.

Purified AqNR2 also bound fatty acids (Figure 3B). It did not, however, activate transcription in the mammalian reporter assay but acted as a very strong repressor of basal transcription, irrespective of the type of serum used (Figure 3A). These results indicate that AqNR2 can repress transcription and, like its ortholog HNF4 and its paralog AqNR1, bind FAs. We cannot rule out the possibility that AqNR2 may have the capacity to activate transcription in the presence of some yet unknown ligand.

### Phylogenetic Reconstruction of the Ancestral NR's Functions

**Figure 5:**
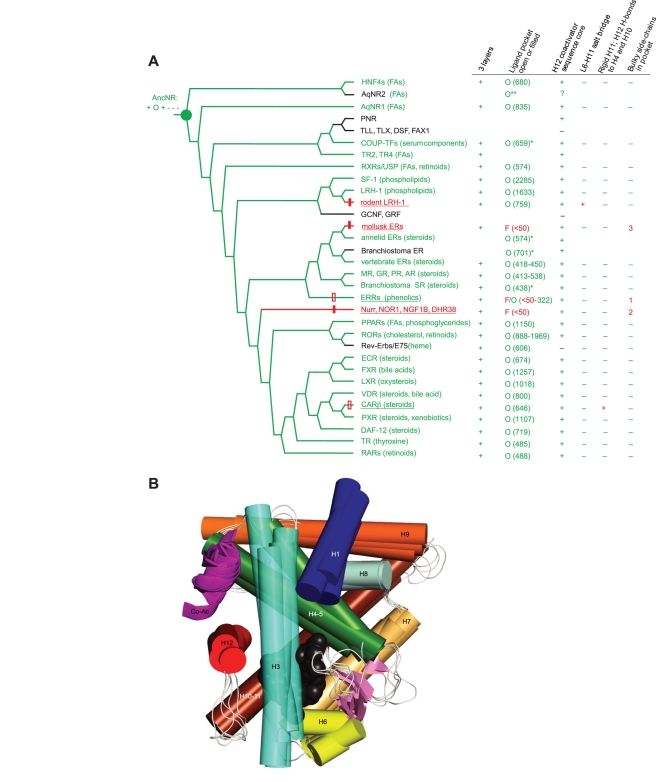
Ancient origin of ligand-activation in the NR superfamily. (A) Reconstruction of structural and functional characters on the NR phylogeny. Ligand-regulated transcriptional activators are shown in green, with ligands in parentheses. Red, activators with no known ligand. Underlined, receptors with transcriptional activity in the absence of ligand or other modifications. Black, repressors that do not activate transcription. The ancestral NR (AncNR, green circle) is shown, with the most parsimonious reconstructions at that node for the characters in the table. Hash marks on branches show gains of ligand-independent activity with or without loss of ligand binding (filled and empty red boxes, respectively). States of protein structural characters are shown in the table. *“3 layers”*: 10 to 12 helices arranged in conserved three-layer sandwich as in panel B. “*Ligand pocket open or filled”*: O, open internal cavity bounded by helices 3, 5, 7, 10, and 12 in crystal structure (cavity volume in Å^3^); O*, open pocket in homology model; O**, open pocket inferred from experimental data on ligand-binding or ligand-regulation; F, filled cavity, <50 Å^3^. “*H12 coactivator sequence”*: presence (+) or absence (−) of canonical co-activator interface φφ*κφφ motif in helix 12 (φ, hydrophobic; *, any residue; κ, charged). *“L6-11 salt bridge*”: presence (+) or absence (−) of salt bridge from the L6-7 loop to helix H10-11. “*Rigid HX and H10-H12 h-bonds*”: presence (+) or absence (−) of short additional helix N-terminal to H12 and hydrogen bonds between H10-11 and H12. “*Bulky residues in pocket”*: presence (1,2,3) or absence (−) of Phe or Trp residues in space occupied by ligand in other receptors; 1, 2, 3 indicate bulky residues at different sets of sequence sites (see Figure 6). Blank cells, no data available. Rodent LRH-1 has been coded as a ligand-independent activator based on its crystal structure, but functional assays have not established its ligand-independence. (B) Shared structural basis for ligand-dependent activation in NR LBDs. The peptide backbones and ligands are superimposed for five distantly related NRs: human HNF4α, mouse RXRα, human ERα, mouse RARβ, and human PPARα. Gray spheres show ligands. Helices (shown as cylinders) are numbered, and the coactivator peptide (Co-Ac) is a magenta ribbon. The beta-sheet between H5 and H6 is pink. The region between H1 and H3, which is structurally variable, has been removed to show the ligands more clearly. For details and PDB identifiers, see Tables S4, S5, and S7.

A robust rooted protein family phylogeny, functional data on basally branching receptors, and recently gathered information on the functions and structures of NRs from model and non-model organisms allow us to infer the characteristics of the ancestral NR. Although the sequences of the NR superfamily are too divergent to allow unambiguous reconstruction of the ancestral NR LBD at the amino acid level, there is substantial phylogenetic signal in the structural and functional features of NR LBDs. We coded these features as discrete phylogenetic characters and reconstructed the best-supported ancestral states using phylogenetic methods (Figure 5A). The ancestral NR (AncNR) is decisively reconstructed as having had the capacity to activate transcription, bind a ligand, and be activated by that ligand. The vast majority of extant NRs, including those in the basal lineages, have these characteristics. The handful of exceptions—ligand-independent activators and pure repressors—are scattered across the tree and are in most cases nested deep within groups of liganded activators, indicating that these states are almost certainly derived. The fact that some ligand-independent receptors bind ligands, which can up- or down-regulate their baseline activity [6]–[8], further supports the reconstruction of the ancestor as having possessed these features. No ligand-independent activators are present in the basally branching NR clades.

When the evolution of these functional characters is traced on the NR phylogeny, the hypothesis of a ligand-binding and ligand-activated AncNR is by far the most parsimonious reconstruction. This scenario explains the characteristics of the entire NR superfamily with only five losses of dependence on ligand. Three of these losses were accompanied by a loss of ligand-binding; in the other two instances (the ERRs and constitutive androstane receptor, CAR), receptors evolved “constitutive” transcriptional activity but retained the ancestral capacity to bind ligands, which regulate that baseline activity (Figure 5A). In contrast, the alternative hypothesis of a ligand-independent AncNR would require both ligand-binding and dependence on the ligand for activation to have been gained 12 independent times, plus a subsequent reacquisition of ligand-independent activity in one lineage (Figure S8). Reconstruction of these characters on the alternate phylogeny that places AqNR1 and AqNR2 as sponge-specific duplicates causes no change in the support for an ancestral liganded-activated receptor vis-à-vis the “constitutive ancestor” hypotheses (Figure S9).

It is also clear that AncNR had the capacity to activate transcription rather than acting as a pure repressor. An ancestral activator requires five losses of activity in the lineages leading to the inactive repressor NRs (Figure 5A), whereas 11 independent gains of transcriptional activity would be required if AncNR were transcriptionally inactive.

### Phylogenetic Reconstruction of the Ancestral NR's Structure

A key element of assessing homology is to determine whether shared structures and mechanisms underlie apparently similar features in different lineages. To further test the hypothesis that ligand-binding and activation are homologous functions derived from the ancestral NR—and that ligand-independent activation was repeatedly derived—we analyzed the underlying structural mechanisms for these functions in a phylogenetic framework. We coded as discrete phylogenetic characters the relevant structural features of NR LBDs and phylogenetically reconstructed the best supported ancestral state for each (Figure 5A).

AncNR is decisively reconstructed as having had the shared features of extant ligand-activated receptors that underlie ligand binding and activation. Specifically, there is strong support for the ancestor having possessed (1) the classic NR fold in the active conformation consisting of three layers of helices in highly conserved positions; (2) an open ligand pocket with volume of at least 300 Å^3^, bordered by helices H3, H4-5, H7, H10, and H12; and (3) a surface for binding coactivator proteins, composed of residues in the ligand-stabilized helices H3, H4-5, and H12, with a conserved coactivator-recognition motif in the latter [5],[26],[27]. These features and a similar location of the ligand within a highly conserved LBD structure are shared by even the most distantly related ligand-activated NRs (Figure 5B); indeed, even the ligand-independent receptors retain some or all of these features.

The identical structural basis for ligand-activation throughout the superfamily provides strong evidence that this function is derived from the common NR ancestor. It is plausible that the ancestral ligand was an FA, because several of the most basal lineages bind FAs. Further, the key hydrogen bond between the FA's carboxyl-group oxygen and the Arg side chain on helix 5 is conserved in several basal lineages, including HNF4s, RXRs, and AqNR1. The ligand that historically activated AncNR could have been a ubiquitous endogenous molecule that served as a receptor cofactor, a hormone-like regulatory compound endogenously produced under specific conditions, or an exogenous nutrient or other substance, such as fatty acids produced by bacteria or other species.

**Figure 6:**
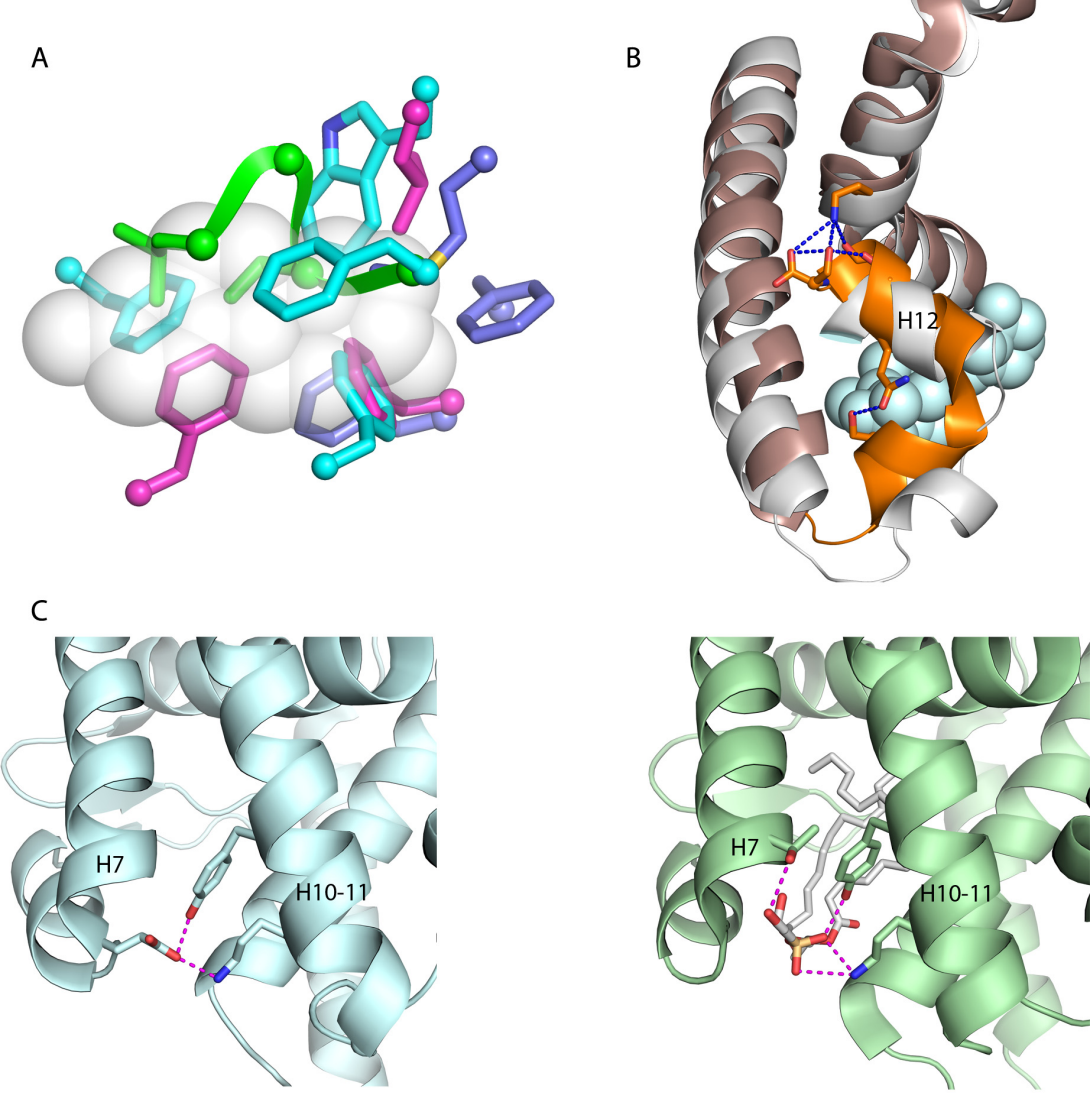
Independent structural mechanisms for ligand-independent activation in nuclear receptors. (A) Receptors with filled ligand cavities. Bulky side chains (shown as sticks) that fill the cavity are shown for rat Nurr1 (cyan), human ERRα (magenta), and oyster ER (blue), with α-carbons as balls. Green ribbon, backbone of the loop with side chains that fill the pocket in *D. melanogaster* Ftz-F1. The van der Waals space occupied by estradiol in human ERα is in white. (B) In human CAR (bronze), a network of hydrogen bonds (blue) stabilizes the activation-function helix H12 and a novel adjacent helix (orange). These interactions are absent from other NRs, such as the close paralog VDR (white), shown with its ligand vitamin D (blue spheres). (C) In the LRH-1 protein of rodents (left, blue), a salt bridge and hydrogen bond between side chains on helices 7 and 10 replaces similar interactions in human LRH-1 (right, green) between each helix and the ligand (white). The ligand-mediated interactions and the amino acids involved in human LRH-1 are also present in the closest NR paralog SF-1 [33]. For PDB identifiers, see Table S7.

In contrast, the structural elements that appear to confer ligand-independence differ dramatically among the ligand-independent activators (Figure 6). In Nurr1/DHR38, the mollusk estrogen receptor, and the vertebrate ERRs, the pockets are filled with multiple bulky hydrophobic side chains that mimic the presence of ligand [7],[8],[28],[29], but the sites and states involved in the three receptor classes are all different, with a single convergent exception in two of the three receptors (Figure 6A). In *Drosophila* Ftz-F1, the H6/H7 region adopts an unprecedented loop conformation that turns inward and fills the cavity (Figure 6A). In CAR, residues in helix 12 (H12) form unique hydrogen bonds to H4-5, and a novel helix, absent from other NRs, packs against H12, stabilizing the active conformation (Figure 6B) [30]–[32]. Finally, in the crystal structure of mouse LRH-1, the active conformation is stabilized without ligand due to a unique salt bridge between residues in H7 and H10, which replaces a similar bridge between the ligand and H10 in orthologs of the same protein of humans and other species, and in SF-1, the closest paralogous NR (Figure 6C) [33].

These radical differences in putative underlying mechanisms indicate that ligand-independent activity is a convergent character with independent evolutionary origins rather than a homologous feature inherited from the common NR ancestor. The hypothesis of a ligand-dependent AncNR explains the structure-function relations of the vast majority of present-day receptors as due to descent from an ancestor that possessed those same features. In contrast, the hypothesis of an unliganded AncNR can explain the structure-function relations of only a single NR as due to descent from the ancestral NR; it requires the ancestral basis for ligand-independent activity to have been independently lost and replaced with different underlying mechanisms in all other lineages of ligand-independent receptors and the shared mechanisms for ligand-dependent activation to have been gained independently in the many lineages of liganded receptors.

### How “Novelty” Evolves

Our findings indicate that NR LBDs evolved their functional diversity by tinkering with a ligand-dependent transcriptional activator. Ligand-regulated NRs are thermodynamically tuned so that in the appropriate contexts the active conformation is favored in the presence of activating ligand but not its absence. The most common functional shift during NR evolution was modification of ligand specificity due to subtle changes in the shape and surface properties of the ancestral ligand pocket. Both historical and contemporary studies indicate that such shifts in ligand preference can evolve through a relatively small number of mutations that subtly alter the ligand cavity (e.g., [34]–[36]).

In a few lineages, ligand-independent activity evolved by mutations that stabilized the active conformation in the absence of ligand; in two such cases, the cavity remained open, yielding a receptor whose baseline activity can be antagonized or super-activated by ligands [6],[8]. Laboratory and clinical data contain many examples of ligand-independent activity evolving via single point mutations that add sufficient stability to the active conformation in the absence of ligand (Table S2). Historical studies also document the evolution of constitutive activity with a very simple genetic basis [33]. Such transitions tip the thermodynamic balance so that the formerly switchable LBD becomes stuck in the “on” position, irrespective of ligand. In contrast, evolving a ligand-dependent receptor from a ligand-independent ancestor would require mutations that (1) generate a ligand pocket of the appropriate size and shape to accommodate some ligand and (2) destabilize the active conformation just enough to abolish ligand-independent activity but not so much that the capacity is lost to activate transcription when ligand is present. We observed no such transitions on the NR phylogeny, and we are aware of only one NR mutation that accomplishes this end in the laboratory; that example reflects a return to the ancestral amino acid state in a receptor that binds ligand but also possesses ligand-independent activity [37].

Finally, in a few other lineages, inactive repressor NRs evolved by degradation of the activation function without loss of other functions, such as DNA binding, dimerization, or corepressor binding (see [11]). Indeed, most inactive NRs have simply lost the co-activator interaction motif in H12 but retain the classic LBD secondary and tertiary structure, and some even retain an open pocket [12]. Inactive repressor NRs have been shown to evolve from ligand-activated precursors via simple genetic mechanisms that disable ligand or coactivator binding but leave intact other functions of the receptors, such as DNA binding, dimerization, and corepressor binding [11].

Most gene families, like the NRs, have some common conserved core function—some catalytic activity, for example, or the capacity to interact with DNA. Functional diversity within such families is conferred by members' binding to and carrying out that function on different partners. Our observations in the NRs underscore the capacity of evolution to produce dramatic functional diversity by tinkering with a common ancestral template over long periods of time. The varied and subtle nature of these tinkering events is revealed only when densely sampled structural and functional data are analyzed in a phylogenetic context. We predict that, when sufficient data are gathered to allow detailed evolutionary reconstructions, it will become apparent that most protein superfamilies diversified by subtle modification and partial degradation of ancient, deeply homologous functions. Invoking the evolution of wholesale “novelty” will seldom be necessary.

## Methods

### Alignment and Phylogenetic Analyses

Nuclear receptor protein sequences were obtained by mining the genomes of *Amphimedon queenslandica*, *Trichoplax adhaerens*, *Nematostella vectensis*, *Lottia gigantea*, *Capitella capitata*, and *Branchiostoma floridae* (Table S1). The assembled genomes and developmental expressed sequence tags were screened using tBlastn with LBD and DBD amino acid sequences from each known NR family. Further analysis using PFAM domain analysis (PF00104 and PF00105) [38] and a hidden Markov model-based method (PTHR11865) [39] confirmed the presence of only two NRs in the *A. queenslandica* genome, which has been sequenced at approximately 9-fold coverage [19]. In some of the other genomes, gene model sequences were modified to resolve gaps in the sequence by performing a local assembly with gene traces or to correct the predicted protein sequence based on alignment with other conserved domain sequences. Complete NR complements from the curated whole-genome databases of *H. sapiens*, *D. melanogaster*, *C. intestinalis*, *F. rubripes*, and *S. purpuratus* were also included. Additional nuclear receptors were identified by using the SMART domain-based sequence annotation resource [40] to search the UniPROTKB/TrEMBL database based on the amino acid sequence of the ERR of *Marisa cornuarietis*. Receptors for which only partial sequence was available (missing >20% of the DBD or LBD) and those entirely lacking a DBD domain (e.g., human DAX1 and SHP) or LBD domain (e.g., *D. melanogaster* Knirps and Knrl) were excluded from the analysis.

A total of 275 nuclear receptor sequences were aligned. Full-length sequences containing the DBD, highly variable hinge region, and LBD were aligned using Multiple Sequence Alignment by Log-Expectation (MUSCLE) v. 3.6 [41] in order to identify the boundaries of the conserved regions. After removal of the variable (non-alignable) hinge region, sequence blocks corresponding to the DBD and LBD were then aligned separately using MUSCLE. The DBD and LBD alignments were checked manually to remove lineage-specific indels, and the LBD alignment was checked to ensure correct alignment of the conserved AF-2 core sequence (φφ*κφφ motif; φ, hydrophobic; *, any residue; κ, charged). Amino acids C-terminal to this AF-2 core sequence could not be reliably aligned among all nuclear receptors and the LBD alignment was therefore truncated after the AF-2 core sequence. The DBD and LBD alignments were then concatenated in MacClade 4 (Sinauer Associates, Inc., MA, USA) for subsequent phylogenetic analyses. We then used APDB software [42] to characterize the quality of our alignment with reference to the 26 NR LBDs in the alignment for which X-ray crystallographic structures are available; the average iRMSD (the root mean square difference of the intramolecular distances between aligned pairs of alpha-carbons) over the entire LBD alignment was 0.82 angstroms, well under the resolution of the structures themselves, indicating that the alignment has high structural plausibility.

Phylogenetic analyses were performed using maximum likelihood in PhyML v. 2.4.5 [43] and Bayesian analysis using MrBayes v. 3.1 [44]. The Jones-Taylor-Thornton model with a four-category discrete gamma distribution of among-site rate variation (ASRV) and a proportion of invariant sites was used. For ML, all model parameters were optimized by maximum likelihood. Support was evaluated by obtaining the approximate likelihood ratio for each node—the estimated ratio of the likelihood of the best tree with the split to the best tree without the split [45]—as well as the chi-square confidence metric, which approximates 1−*p*, where *p* is the probability that an approximate likelihood ratio as great or greater than that observed at a resolved node would occur if the null hypothesis of an unresolved node is true [45]. To identify the next best alternative tree for the basal split between the AqNR1 and AqNR2-containing groups, we used Phyml to optimize the branch lengths and model parameters on each of the two possible rearrangements of the ML tree around this internal branch and then report their likelihoods. To determine the effect of fast-evolving sites on the inference of phylogeny, we used PAML software to identify sites in the top two octiles of the gamma distribution (Table S3) and repeated the analysis with those 113 sites removed. To facilitate adequate sampling of tree space in Bayesian analysis [46], we used a 174-sequence taxon-trimmed MUSCLE-aligned dataset including nuclear receptors from the following taxa representative of the major metazoan lineages: *Acropora millepora*, *Nematostella vectensis*, and *Tripedalia cystophora* (cnidarians); *Amphimedon queenslandica* and *Suberites domuncula* (poriferans); *Branchiostoma floridae* (cephalochordate); *Capitella capitata* and *Lottia gigantea* (lophotrochozoans); *Ciona intestinalis* (urochordate); *Drosophila melanogaster* (ecdysozoan); *Homo sapiens* (vertebrate); *Saccoglossus kowalevskii* (hemichordate); *Strongylocentrotus purpuratus* (echindoderm); and *Trichoplax adhaerens* (placozoan). Terminal branches of length ≥0.76 in the PhyML analysis (except for AqNR2) were removed. Four heated chains were run for 8 million generations with temperature 0.3; the cold chain was sampled every 100 generations. Priors were uniform on topologies, uniform (0, 5) on branch lengths, and uniform (0.1, 10) on the alpha shape parameter. The first 6,694,000 generations were discarded as burn-in, because at this point in the chain the standard deviation of posterior probabilities over all splits was <0.01 and the two chains had converged as evaluated using the “compare” option of AWTY software [47]. We also repeated ML analysis on this reduced 174-sequence dataset and found no change in the relationships among NR types (Figure S5).

The phylogeny shows that AqNR1 is the ortholog of the previously identified but misnamed “RXR” gene identified in the sponge *Suberites domuncula* [48] and is identical to the “HNF4” gene previously reported in *A. queenslandica* [49]. To determine the minimum number of gene duplications and losses, we used SDI software [50]. The 275-sequence ML phylogeny was reduced by collapsing sets of orthologous NRs within major taxa—Porifera, Placozoa, Cnidaria, Protostomia, Deuterostomia—into single clades. To avoid spurious inference of duplication/loss due to phylogenetic error, nodes with likelihood-ratio support <10 that conflicted with the accepted taxonomic phylogeny (Porifera, (Placozoa, (Cnidaria, (Protostomia, Deuterostomia)))) were treated as unresolved and reordered to be congruent with the taxonomic phylogeny. SDI software was then used to reconcile this gene family tree with the taxonomic tree and identify the root with the lowest possible mapping cost (duplications plus losses). The mapping cost was also calculated for all possible roots of the gene family phylogeny, except for rootings on branches after the Cnidaria/Bilateria divergence, which have higher mapping costs and were judged to be implausible. Reconstructions of ancestral structural and functional states were performed manually using Fitch parsimony.

### Isolation of Amphimedon Nuclear Receptor Genes

Demosponge *Amphimedon queenslandica* were collected from Heron Island Reef, Great Barrier Reef, and total RNA was isolated from larvae using RNeasy Mini kit (Qiagen, Valencia, CA). The coding regions of AqNR1 and AqNR2 were obtained using BD SMART RACE cDNA Amplification kit (Clontech, Mountain View, CA), and the full reading frames were amplified by RT-PCR, cloned into pGEM-T EASY vector (Promega, Madison, WI), and verified by sequencing. In situ hybridization analysis of RNA expression was conducted as previously described [49].

### Transcriptional Activation Assays

AqNR1, AqNR2, and rat HNF4α receptor LBDs, including the hinge region and carboxy-terminal extension, were amplified by high-fidelity PCR using Phusion DNA polymerase (New England Biolabs, Ipswich, MA) and cloned into a GAL4-DBD-pSG5 expression vector (gift of D. Furlow). AqNR1-LBD (gi ACA04755) consisted of amino acids 263636, and AqNR2-LBD (GU811658) included amino acids 118–852. Rat HNF-4α template was a gift from Frances Sladek; the LBD used consisted of amino acids 116–465 (NP_071516). Site-directed mutagenesis was performed using QuickChange II (Stratagene, La Jolla, CA) and verified by sequencing.

Chinese hamster ovary (CHO-K1) cells were grown in a 96-well plate and transfected with1 ng of receptor plasmid, 100 ng of a UAS-driven firefly luciferase reporter (pFRluc), and 0.1 ng of the constitutive phRLtk *Renilla* luciferase reporter plasmid, using Lipofectamine and Plus Reagent in OPTIMEM (Invitrogen, Carlsbad, CA). After 4 h, transfection medium was replaced with phenol-red-free αMEM supplemented with 10% dextran-charcoal-stripped fetal bovine serum (Hyclone, Logan, UT). Cells were allowed to recover and express protein for 48 h, and then assayed by luminometry using the Dual-Glo luciferase system (Promega, Madison, WI). Firefly luciferase activity was normalized by *Renilla* luciferase activity.

### Protein Expression and Purification

AqNR1 LBD (residues 415–636), AqNR1 mutant proteins, AqNR2 LBD (residues 616–852), and rHNF4α (residues 133–382) were expressed as N-terminal hexahistidine maltose binding protein fusions with a TEV cleavable linker in pLIC-MBP (a gift from J. Sondek) and grown in *E. coli* BL21(DE3) pLysS cells using standard methods. Protein was purified using affinity chromatography using standard methods. Following TEV cleavage, the resulting 6xHis-tagged MBP was removed using an additional nickel affinity column and the AqNR1 or AqNR2 was polished via gel filtration. Pure AqNR1 or AqNR2 was dialyzed against 150 mM ammonium acetate (pH 7.4) prior to lipid extraction.

### Mass Spectrometry

Organic solvent extraction was performed on purified LBDs from bacteria to facilitate detailed characterization of bound ligands in the absence of protein. Before extraction, 0.1 mg of C13 labeled palimitic acid was added as an internal standard. Lipid from approximately 4 mg of wild-type or mutant forms of AqNR1 LBD, AqNR2, or rHNF-4 LBD were extracted with a 2∶1 chloroform/methanol (v/v) solution and then analyzed by negative ion ESI/MS. All extractions were performed in duplicate. Mass spectra were acquired on a LTQ FT Hybrid Mass spectrometer (Thermo Finnigan LTQ-FTMS, Somerset, NJ) equipped with an electrospray source. Typically, 10 µL of the aforementioned lipid solution was diluted into 10 µL water/acetonitrile (2∶1 v/v) and subjected to ESI/MS in the negative ion mode. In addition to the fatty acids shown in Figure 2B, an additional unidentified substance at ∼421 m/z was also bound when AqNR1 was incubated with either complete or stripped serum. All samples were run in triplicate. Data acquisition and analysis were performed using the instrument's xcalibur software.

### Free Fatty Acid Binding

Purified wild-type or mutant hexahistidine maltose binding protein fused AqNR1 was incubated with undiluted complete (Invitrogen - 26010) or cyclodextran/charcoal stripped (HyClone -SH30068.03, Waltham, MA) serum at a ratio of 20 mg protein to 5 ml undiluted serum. The protein/serum mixture was incubated overnight at 4°C followed by re-purification over a nickel affinity column. Protein purity was assessed by SDS-PAGE and fractions containing pure wt or mutant AqNR1 were pooled. Bound lipids were then quantified using the free fatty acid quantification kit from BioVison Inc. (Mountain View, CA). 0.5 mg of each purified LBD was subject to chloroform/detergent extraction to isolate the long chain free fatty acids. Extracted fatty acids were enzymatically converted to their CoA derivatives and oxidized, allowing quantitation in a colorimetric assay (λ = 570) relative to a standard curve generated using palmitic acid.

### Homology Modeling and Calculations of Cavity Volumes

Efforts to determine the crystal structure of AqNR1-LBD were unsuccessful, so its structure was predicted by homology modeling and energy minimization. The AqNR1 LBD amino acid sequence was aligned to and threaded on human HNF-4α (PDB 1M7W) and then energy minimized with palmitic acid—the most abundant experimentally bound ligand—using the Homology module in InsightII (Accelrys, Inc., San Diego, CA).

To calculate ligand pocket volumes of receptors with X-ray crystal structures, we used VOIDOO [51] in probe-occupied mode. We assigned the centroid of the bound ligand or a manually defined point as a starting locus for cavity searches. Cavity volumes were calculated using 10 random orientations of the protein using 10 different “van der Waals growth factors” ranging from 1.1–1.3. Mean and mode cavity volumes with standard deviation are listed in Table S4.

To calculate ligand pocket volumes of receptors whose structures have not yet been determined by x-ray crystallography, we inferred homology models. Specifically, we created homology models of the LBDs of AqNR1 (gi 167859601, residues 404–534), annelid ER (186908731, residues 231–479), Branchiostoma SR (170178459, residues 298–532), and Branchiostoma ER (170178461, residues 250–504). In each case, we used as templates crystal structures of several NR LBDs with a variety of cavity volumes, including human ERα with estradiol (PDB 1ERE:A, cavity volume 447 Å^3^), human ERR3 apo form (1KV6:A, cavity volume 262 Å^3^), human ERR1 apo form (3D24:A, cavity volume 42 Å^3^), and AqNR1 as modeled on template HNF4A with DAO (1MV7:A, cavity volume 680 Å^3^). We generated 10 models for every protein with Modeller 9.7 using the default parameters [52]. Models were visually inspected for artifacts (e.g., knotting) and further assessed using RamPage in CCP4i software; only models with 95% of residues in the preferred region and <1% of residues in the outlier region of the Ramachandran map were accepted. We then used Voidoo software as described above to calculate cavity volumes, which are listed in Table S5.

## Supporting Information

Figure S1 **Expression of AqNR1 and AqNR2 during sponge development, visualized using in situ hybridization.**
(0.49 MB PDF)

Figure S2 **Maximum likelihood phylogeny of the nuclear receptor superfamily.**
(0.33 MB PDF)

Figure S3 **Phylogeny of the nuclear receptor family inferred using Bayesian MCMC.**
(1.24 MB PDF)

Figure S4 **Maximum likelihood NR phylogeny with fast-evolving sites removed.**
(0.81 MB PDF)

Figure S5 **Maximum likelihood NR phylogeny on a dataset with reduced taxon sampling.**
(0.68 MB PDF)

Figure S6 **Next-best phylogenetic arrangement of the basal split between sponge NR paralogs.**
(0.61 MB PDF)

Figure S7 **Profile of lipids associated with AqNR1, detected using mass spectrometry.**
(0.65 MB PDF)

Figure S8 **Reconstruction of NR ligand-binding and evolution if AncNR is assumed to have been a ligand-independent activator.**
(0.54 MB PDF)

Figure S9 **Reconstruction of ancestral structural and functional characters on an alternate NR phylogeny.**
(0.58 MB PDF)

Table S1 **Species, taxonomic classification, abbreviations, and accession numbers of NR sequences used in phylogenetic analyses.**
(2.00 MB PDF)

Table S2 **Mutations known to cause constitutive activity in liganded NRs or ligand-activation in constitutive receptors.**
(0.18 MB PDF)

Table S3 **Sites in NR alignment classified by evolutionary rate.**
(1.10 MB PDF)

Table S4 **Protein structures and ligands used for calculating cavity volumes.**
(0.53 MB PDF)

Table S5 **Cavity volumes of modeled nuclear receptors using different templates**
(0.75 MB PDF)

Table S6 **Key to nuclear receptor nomenclature.**
(0.44 MB PDF)

Table S7 **Protein data bank accession numbers for structures referred to in the text.**
(0.30 MB PDF)

## Abbreviations

AncNR: ancestral NR
CAR: constitutive androstane receptor
DBD: DNA-binding domain
FA: fatty acid
LBD: ligand-binding domain
