# Supplementary material for: Protein Evolution by Molecular Tinkering: Diversification of the Nuclear Receptor Superfamily from a Ligand-Dependent Ancestor
Source: PLoS Biol. 2010 Oct 5;8(10):e1000497. doi: 10.1371/journal.pbio.1000497 (PMC2950128; doi:10.1371/journal.pbio.1000497)
Supplement: Figure S1 — Expression of AqNR1 and AqNR2 during sponge development, visualized using in situ hybridization. (0.49 MB PDF) [file pbio.1000497.s001.pdf]

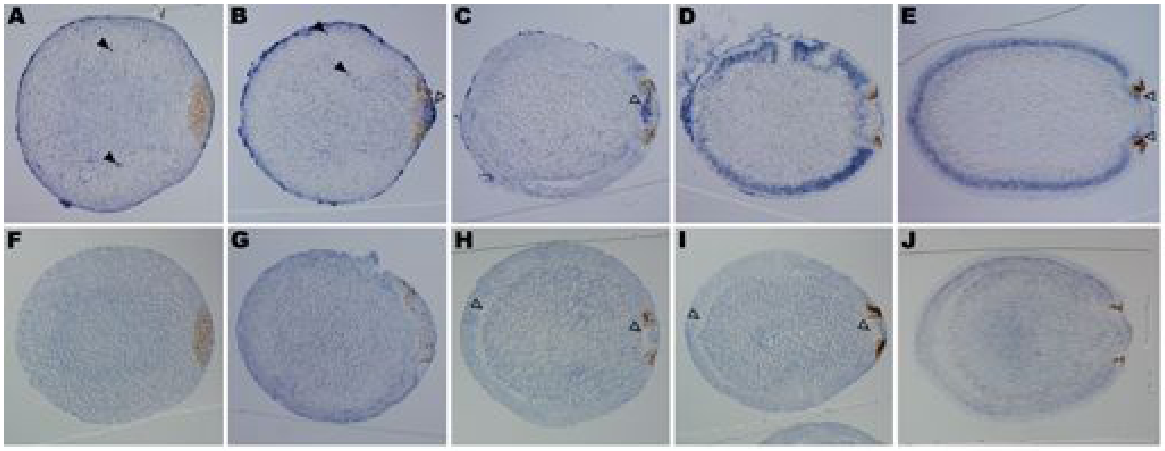

Fig. S1. Expression of AqNR1 (A-E) and AqNR2 (F-J) during sponge development.
In situ hybridization was performed as previously described (Larroux et al 2006). AqNR1 transcripts are detected in a small subset of cells of the outer layer after gastrulation (black arrowheads in A and B) as well as in the outermost region of the embryo and in the maternal follicle cells. When the pigment spot is forming, a strong expression domain appears inside the ring (empty arrowhead in B). Once the ring is formed, this domain appears to shift to the basal region of large cells that form the outer layer of the posterior pole (empty arrowhead in C). At this stage, expression persists in the follicle layer expression. AqNR1 is expressed in the columnar epithelial layer of the elongated embryo before it hatches (D), as in the larva (E; Larroux et al 2006). AqNR1 transcripts are also detected in the basal region of cells associated with the pigment ring (empty arrowheads in E). In contrast, AqNR2 expression appears to be relatively ubiquitous during development (F-J), with the exception of the developing sub-epithelial layer and the basal region of the posterior cells (empty arrowheads in H and I).

Larroux, C, Fahey, B, Liubicich, D, Hinman, VF, Gauthier, M, Gongora, M, Green, K, Worheide, G, Leys, SP, Degnan, BM. Developmental expression of transcription factor genes in a demosponge: insights into the origin of metazoan multicellularity. *Evolution & Development* **8**, 150-173 (2006)
