# Supplementary material for: Protein Evolution by Molecular Tinkering: Diversification of the Nuclear Receptor Superfamily from a Ligand-Dependent Ancestor
Source: PLoS Biol. 2010 Oct 5;8(10):e1000497. doi: 10.1371/journal.pbio.1000497 (PMC2950128; doi:10.1371/journal.pbio.1000497)
Supplement: Figure S2 — Maximum likelihood phylogeny of the nuclear receptor superfamily. (0.33 MB PDF) [file pbio.1000497.s002.pdf]

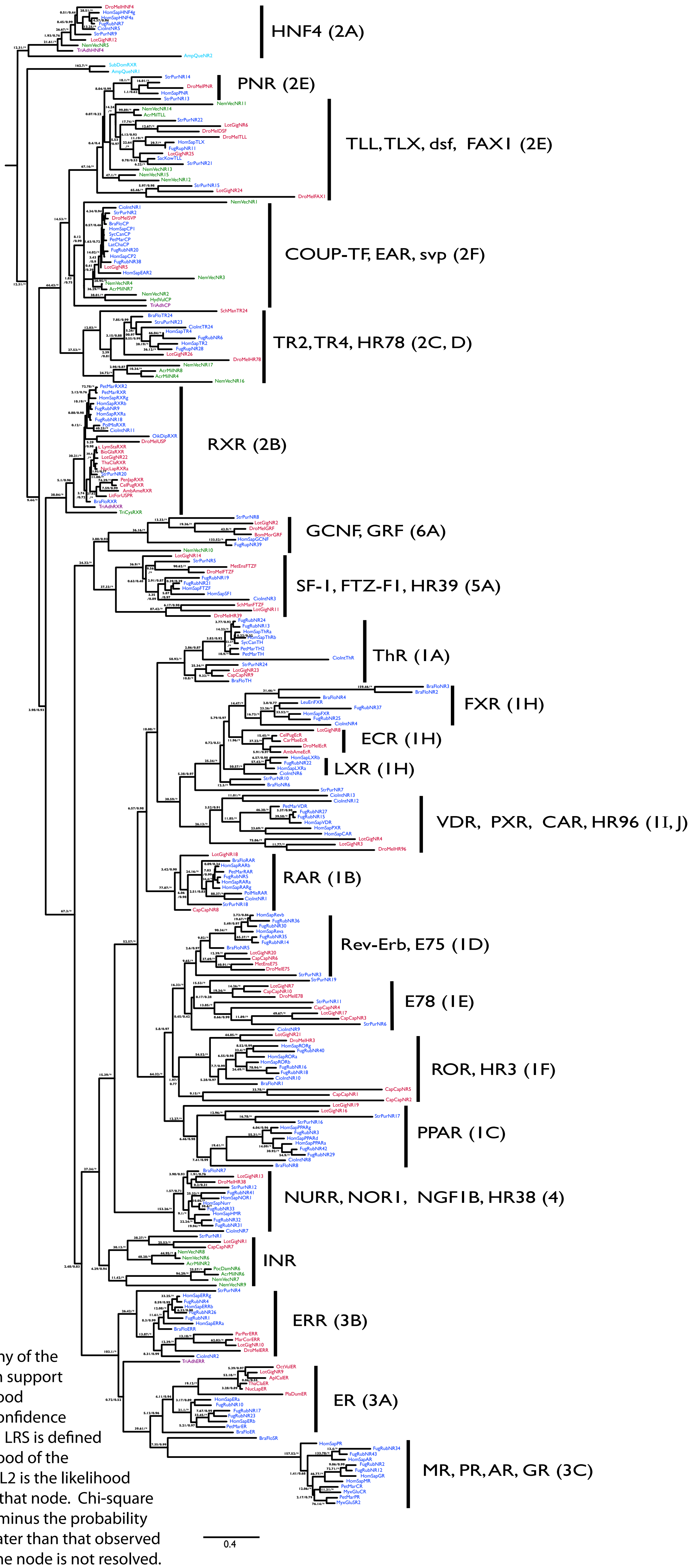

Fig. S2 Maximum likelihood phylogeny of the nuclear receptor superfamily. Branch support is indicated with approximate likelihood ratio statistics (LRS) and chi-square confidence estimates, both calculated in PhyML. LRS is defined as 2*ln(L1/L2), where L1 is the likelihood of the maximum likelihood phylogeny and L2 is the likelihood of the most likely phylogeny lacking that node. Chi-square confidence estimates represent one minus the probability that a likelihood ratio as great or greater than that observed at a node would occur by chance if the node is not resolved. Sequence names are color-coded by taxon: porifera, light blue; placozoan, purple; cnidarian, green; protostome, red; deuterostome, dark blue.
