# Supplementary material for: Protein Evolution by Molecular Tinkering: Diversification of the Nuclear Receptor Superfamily from a Ligand-Dependent Ancestor
Source: PLoS Biol. 2010 Oct 5;8(10):e1000497. doi: 10.1371/journal.pbio.1000497 (PMC2950128; doi:10.1371/journal.pbio.1000497)
Supplement: Figure S3 — Phylogeny of the nuclear receptor family inferred using Bayesian MCMC. (1.24 MB PDF) [file pbio.1000497.s003.pdf]

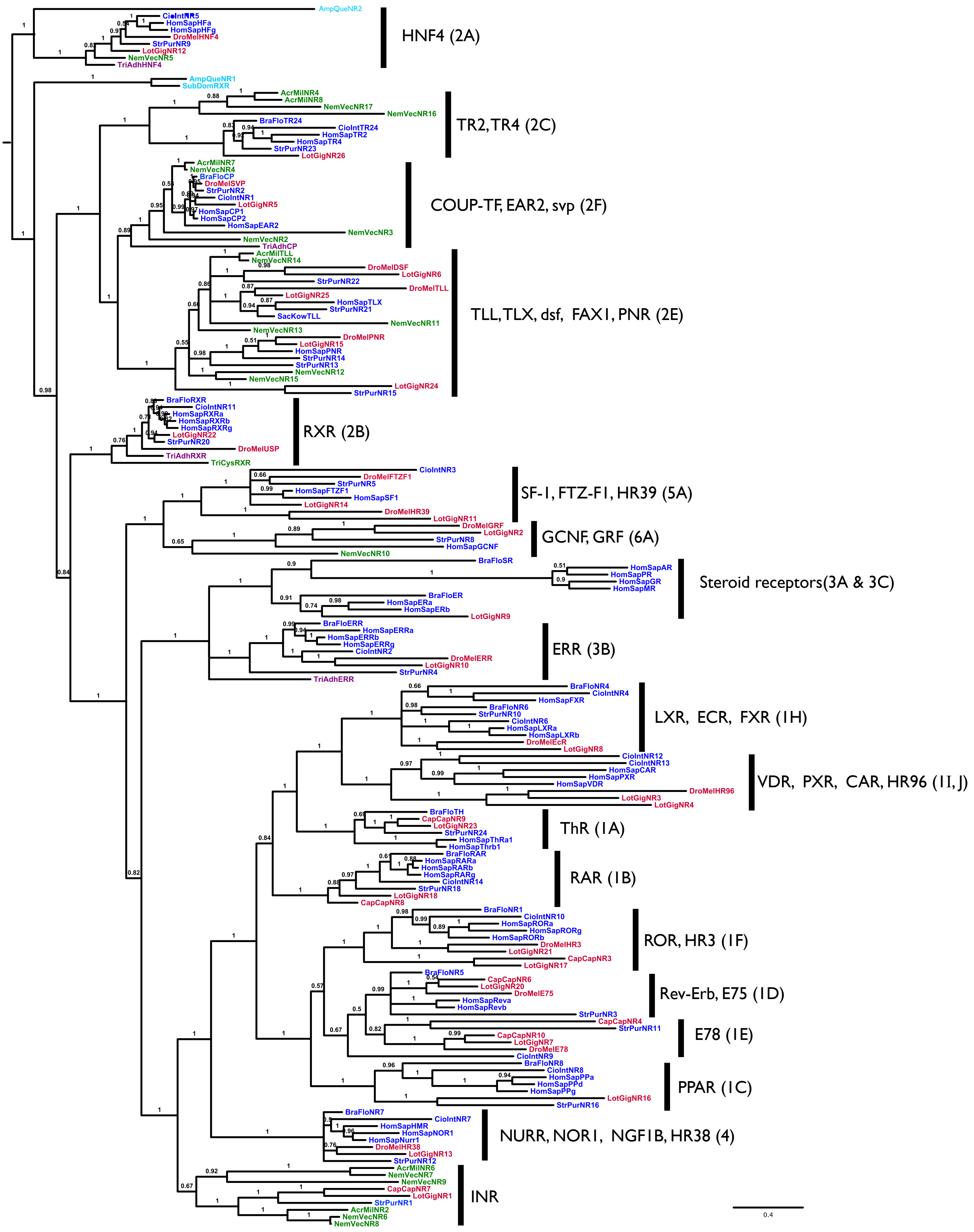

Fig. S3. Bayesian phylogeny of the nuclear receptor family inferred using Bayesian MCMC. Node labels indicate Bayesian posterior probabilities > 50%. Sequence names are colored as in Fig. S2.
