# Supplementary material for: Protein Evolution by Molecular Tinkering: Diversification of the Nuclear Receptor Superfamily from a Ligand-Dependent Ancestor
Source: PLoS Biol. 2010 Oct 5;8(10):e1000497. doi: 10.1371/journal.pbio.1000497 (PMC2950128; doi:10.1371/journal.pbio.1000497)
Supplement: Figure S4 — Maximum likelihood NR phylogeny with fast-evolving sites removed. (0.81 MB PDF) [file pbio.1000497.s004.pdf]

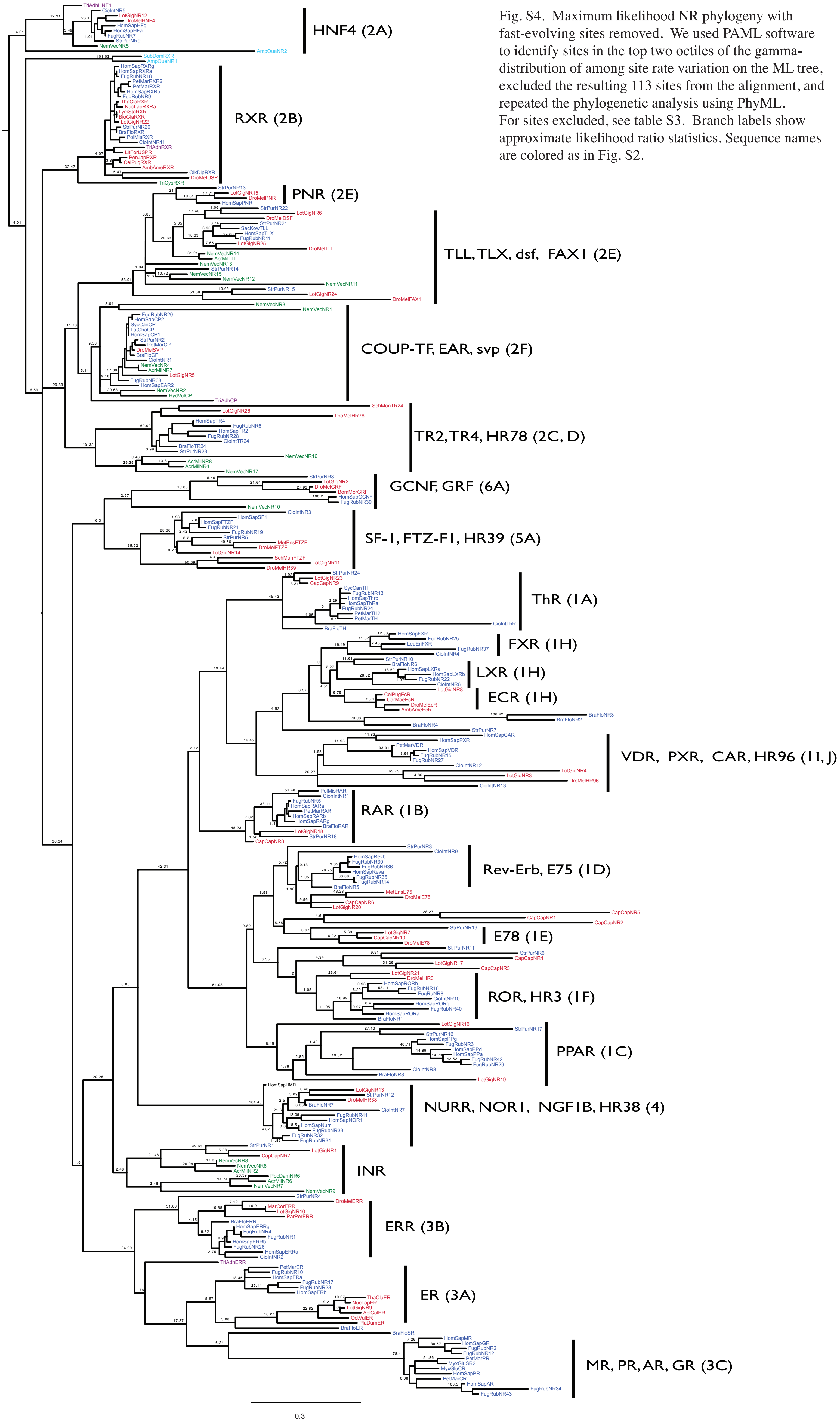

Fig. S4. Maximum likelihood NR phylogeny with fast-evolving sites removed. We used PAML software to identify sites in the top two octiles of the gamma-distribution of among site rate variation on the ML tree, excluded the resulting 113 sites from the alignment, and repeated the phylogenetic analysis using PhyML. For sites excluded, see table S3. Branch labels show approximate likelihood ratio statistics. Sequence names are colored as in Fig. S2.
