# Supplementary material for: Protein Evolution by Molecular Tinkering: Diversification of the Nuclear Receptor Superfamily from a Ligand-Dependent Ancestor
Source: PLoS Biol. 2010 Oct 5;8(10):e1000497. doi: 10.1371/journal.pbio.1000497 (PMC2950128; doi:10.1371/journal.pbio.1000497)

Fig. S5. Maximum likelihood NR phylogeny on a reduced dataset. We used maximum likelihood to analyze the same 174-sequence dataset used for Bayesian MCMC analysis, which excludes the taxa at the end of the longest branches in the 275-sequence dataset. Branch labels show approximate likelihood ratios. Sequence names are colored as in Fig. S2.

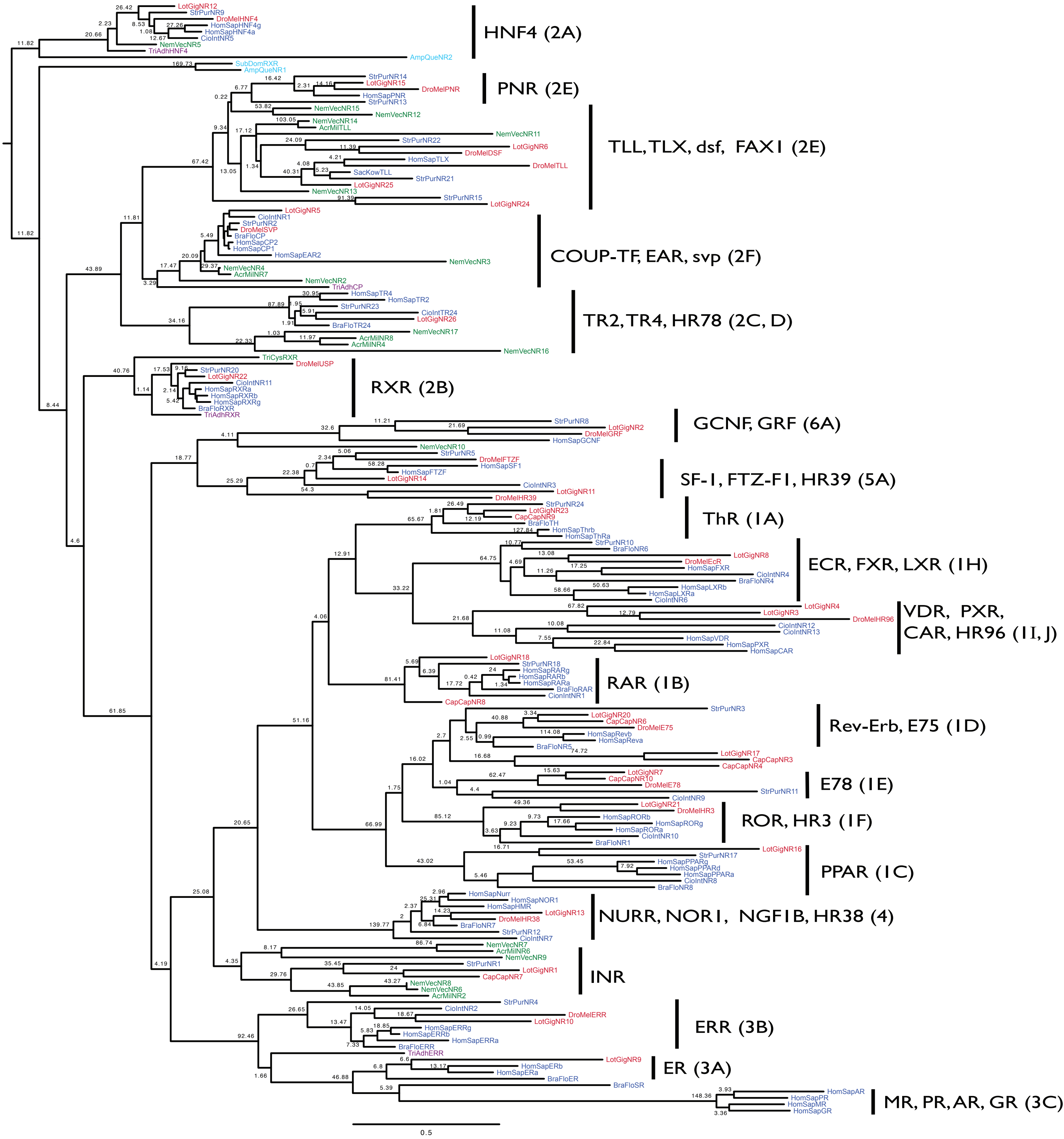

Supplement: Figure S5 — Maximum likelihood NR phylogeny on a dataset with reduced taxon sampling. (0.68 MB PDF) [file pbio.1000497.s005.pdf]
