# Supplementary material for: Protein Evolution by Molecular Tinkering: Diversification of the Nuclear Receptor Superfamily from a Ligand-Dependent Ancestor
Source: PLoS Biol. 2010 Oct 5;8(10):e1000497. doi: 10.1371/journal.pbio.1000497 (PMC2950128; doi:10.1371/journal.pbio.1000497)

Fig. S6. Next-best rearrangement of the basal split in the ML tree that divides sponge NR2+HNF4s from sponge NR1s+all other nuclear receptors. There are three possible topological arrangements of these four groups. Of these, the ML tree in Fig. S2 has the highest likelihood (lnL=-76791.75); the tree with the second highest likelihood (-76799.48) is shown below. The tree below, which unites sponge NR1 and sponge NR2 receptors (blue) to the exclusion of all metazoan NRs, implies a sponge-specific gene duplication (white circle) and an initial duplication (black circle) in the eumetazoan lineage that separated the HNF4 class from other NRs. The speciation event that split sponges from eumetazoa is marked with a white box. The tree is rooted to minimize gene duplications and losses.

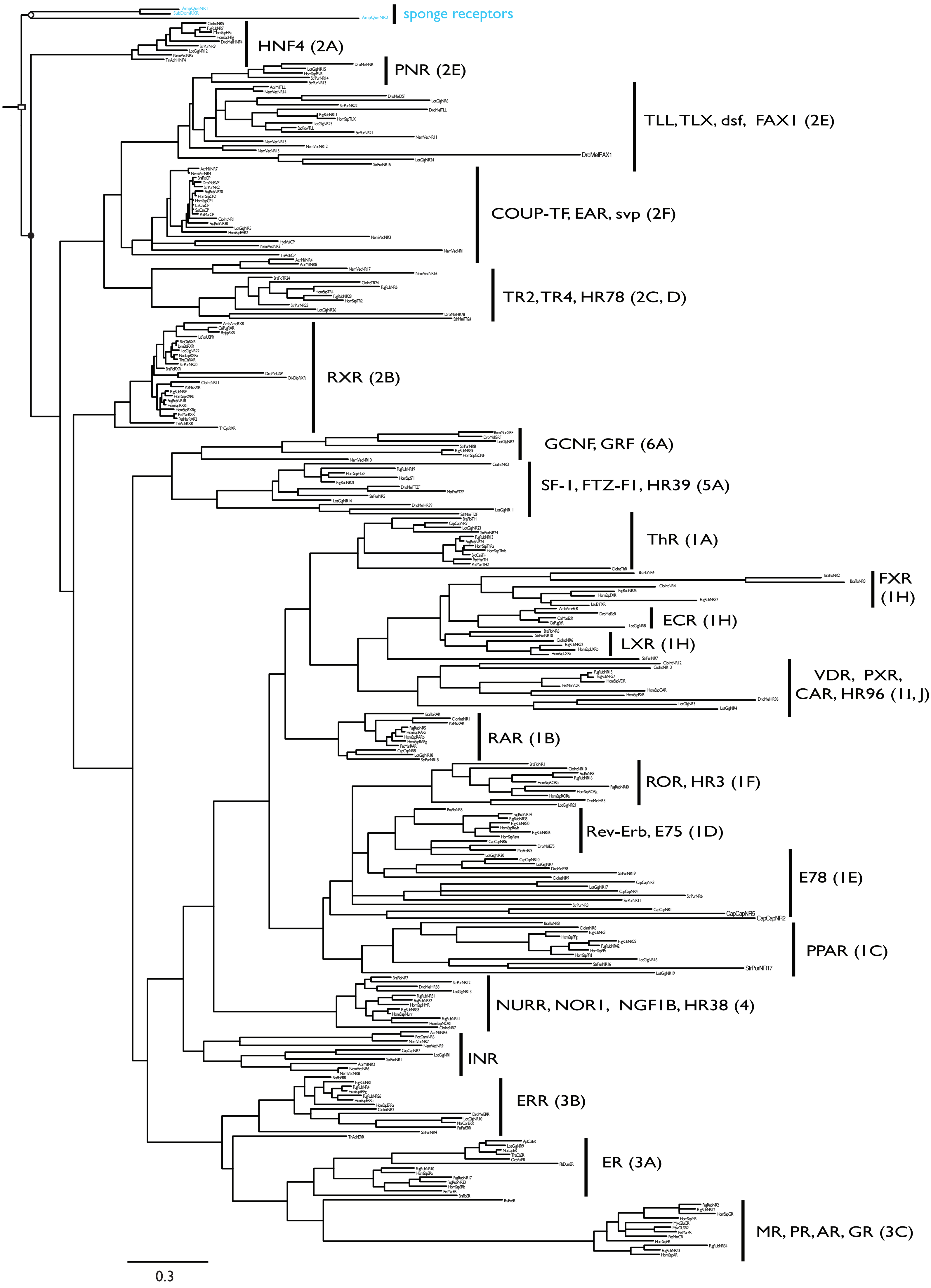

Supplement: Figure S6 — Next-best phylogenetic arrangement of the basal split between sponge NR paralogs. (0.61 MB PDF) [file pbio.1000497.s006.pdf]
