# Supplementary material for: Protein Evolution by Molecular Tinkering: Diversification of the Nuclear Receptor Superfamily from a Ligand-Dependent Ancestor
Source: PLoS Biol. 2010 Oct 5;8(10):e1000497. doi: 10.1371/journal.pbio.1000497 (PMC2950128; doi:10.1371/journal.pbio.1000497)
Supplement: Figure S7 — Profile of lipids associated with AqNR1, detected using mass spectrometry. (0.65 MB PDF) [file pbio.1000497.s007.pdf]

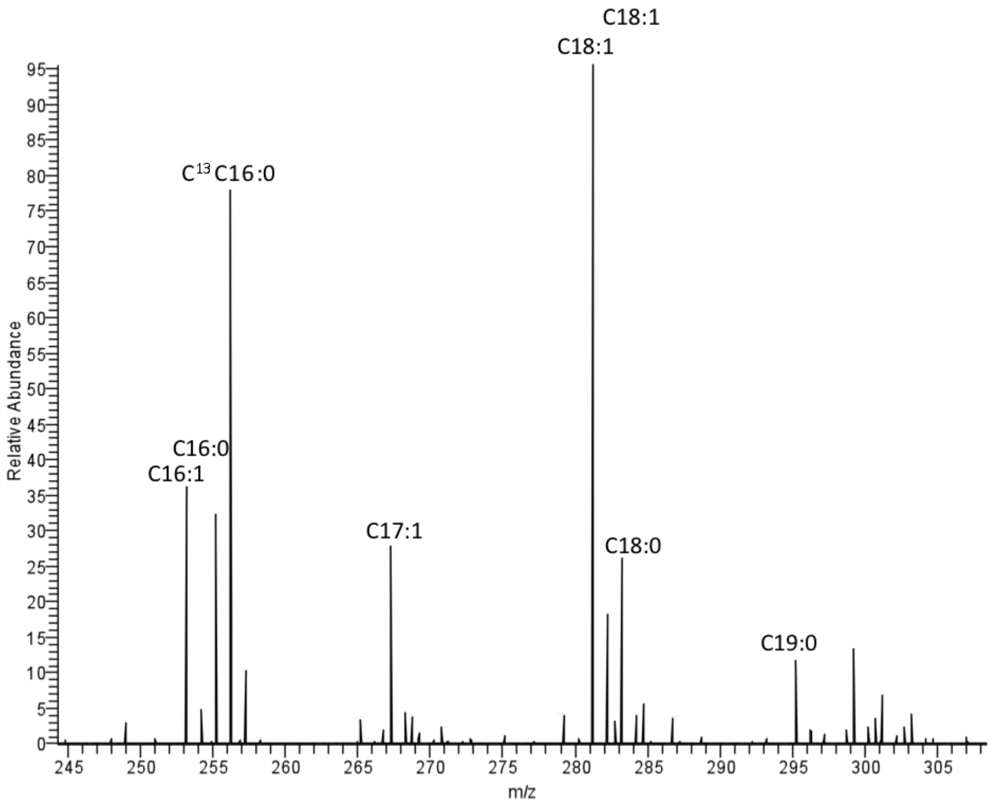

Fig. S7. Profile of lipids associated with AqNR1 as detected by mass spectrometry *Negative Ion ESI/MS of Solvent Extracted AqNR1*. Organic solvent extraction was performed on purified LBDs from bacteria to facilitate detailed characterization of bound ligands in the absence of protein. Before extraction, 0.1 mg of C13 labeled palmitic acid (Cambridge Isotope Laboratories, Inc., Andover, MA) was added as an internal standard. Lipid from approximately 4 mg of wild-type of AqNR1 LBD were extracted with a 2:1 chloroform/methanol (v/v) solution and then analyzed by negative ion ESI/MS. All extractions were performed in duplicate. Mass spectra were acquired on a LTQ FT Hybrid Mass spectrometer (Thermo Finnigan LTQ-FTMS, Somerset, NJ) equipped with an electrospray source. Typically, 10 μL of the aforementioned lipid solution was diluted into 10 μL water/acetonitrile (2:1 v/v) and subjected to ESI/MS in the negative ion mode. Data acquisition and analysis were performed using the instrument's xcalibur software. Fatty acids were identified based on the predicted empirical formula of each peak.
