# Supplementary material for: Protein Evolution by Molecular Tinkering: Diversification of the Nuclear Receptor Superfamily from a Ligand-Dependent Ancestor
Source: PLoS Biol. 2010 Oct 5;8(10):e1000497. doi: 10.1371/journal.pbio.1000497 (PMC2950128; doi:10.1371/journal.pbio.1000497)
Supplement: Figure S8 — Reconstruction of NR ligand-binding and evolution if AncNR is assumed to have been a ligand-independent activator. (0.54 MB PDF) [file pbio.1000497.s008.pdf]

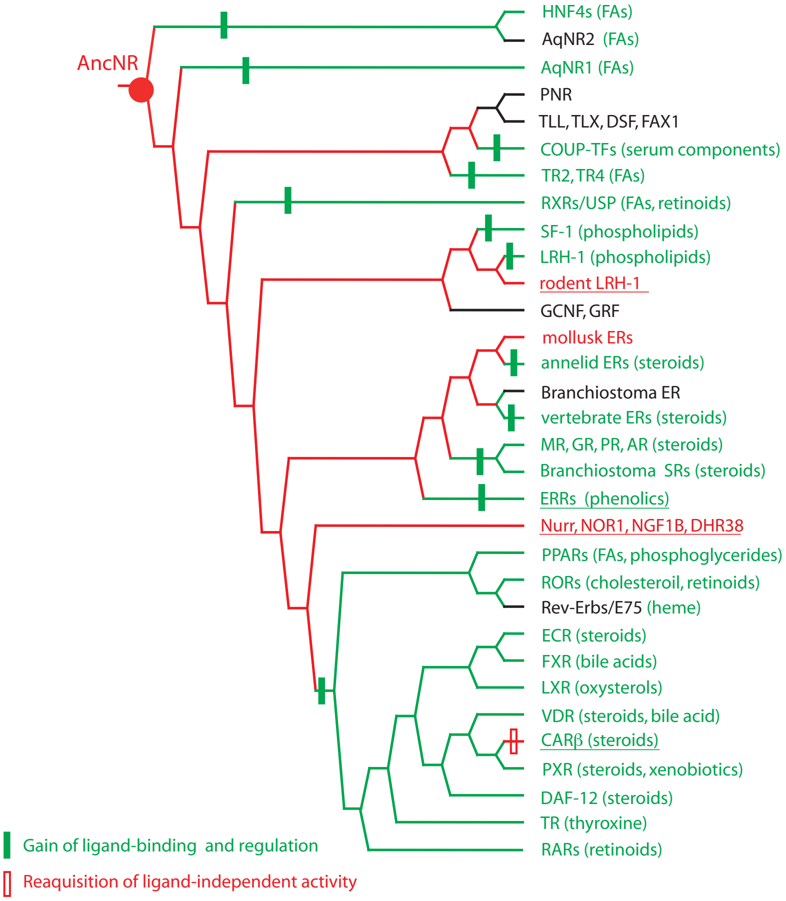

Receptor	3 layers	Ligand pocket open or filled	H12 coactivator sequence core	L6-H11 salt bridge	Rigid H11; H12 H-bonds to H4 and H10	Bulky side-chains in pocket
HNF4s (FAs)	+	O (680)	+	–	–	–
AqNR2 (FAs)		O**	?			
AqNR1 (FAs)	+	O (835)	+	–	–	–
PNR			+			
TLL, TLX, DSF, FAX1			–			
COUP-TFs (serum components)	+	O (659)*	+	–	–	–
TR2, TR4 (FAs)	+		+			
RXRs/USP (FAs, retinoids)	+	O (574)	+	–	–	–
SF-1 (phospholipids)	+	O (2285)	+	+	–	–
LRH-1 (phospholipids)	+	O (1633)	+	–	–	–
rodent LRH-1	+	O (759)	+	–	–	–
GCNF, GRF			–			
mollusk ERs	+	F (<50)	+	–	–	3
annelid ERs (steroids)		O (574)*	+			
Branchiostoma ER		O (701)*	+			
vertebrate ERs (steroids)	+	O (418-450)	+	–	–	–
MR, GR, PR, AR (steroids)	+	O (413-538)	+	–	–	–
Branchiostoma SRs (steroids)	+	O (438)*	+	–	–	–
ERRs (phenolics)	+	F/O (<50-322)	+	–	–	1
Nurr, NOR1, NGF1B, DHR38	+	F (<50)	+	–	–	2
PPARs (FAs, phosphoglycerides)	+	O (1150)	+	–	–	–
RORs (cholesteroil, retinoids)	+	O (888-1969)	+	–	–	–
Rev-Erbs/E75 (heme)	+	O (606)	–	–	–	–
ECR (steroids)	+	O (674)	+	–	–	–
FXR (bile acids)	+	O (1257)	+	–	–	–
LXR (oxysterols)	+	O (1018)	+	–	–	–
VDR (steroids, bile acid)	+	O (800)	+	–	–	–
CARβ (steroids)	+	O (646)	+	–	+	–
PXR (steroids, xenobiotics)	+	O (1107)	+	–	–	–
DAF-12 (steroids)	+	O (719)	+	–	–	–
TR (thyroxine)	+	O (485)	+	–	–	–
RARs (retinoids)	+	O (488)	+	–	–	–

Fig. S8. Reconstruction of NR ligand-binding and evolution if AncNR were a ligand-independent activator. As in Fig. 4, extant receptors are labeled according to their functional characteristics. Ligand-regulated transcriptional activators are shown in green, with ligands in parentheses. Activators with no known ligand are red. Receptors with constitutive transcriptional activity are underlined. Repressors that do not activate transcription are black. Green boxes show gains of ligand-binding and ligand-dependence required if the ancestral NR (AncNR) did not bind a ligand; red box shows reacquisition of ligand-independence. The scenario shown represents the minimum number of evolutionary character changes assuming that extant ligand-independent activators (except CARβ) inherit that activity from the ancestral NR. If CARβ's ligand-independence is also assumed to be inherited from the ancestral NR, an additional 6 gains of ligand-binding and regulation are required.
