# Supplementary material for: Protein Evolution by Molecular Tinkering: Diversification of the Nuclear Receptor Superfamily from a Ligand-Dependent Ancestor
Source: PLoS Biol. 2010 Oct 5;8(10):e1000497. doi: 10.1371/journal.pbio.1000497 (PMC2950128; doi:10.1371/journal.pbio.1000497)
Supplement: Figure S9 — Reconstruction of ancestral structural and functional characters on an alternate NR phylogeny. (0.58 MB PDF) [file pbio.1000497.s009.pdf]

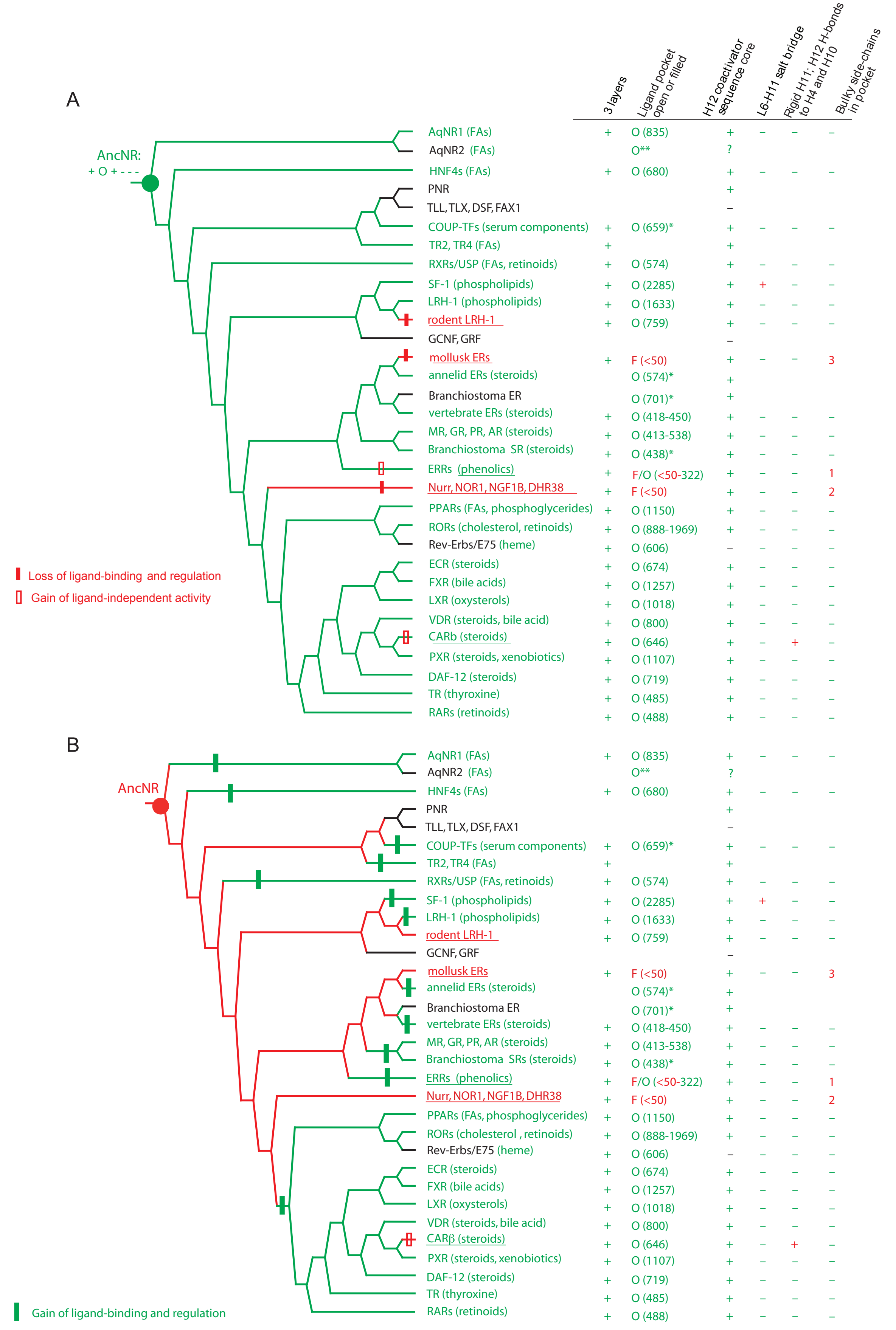

Fig. S9. Reconstruction of structural and functional characters on an alternate NR phylogeny. This phylogeny represents the next-best alternative to the ML phylogeny; it differs in that it groups AqNR1 and AqNR2 as sponge-specific duplicates. A. Maximum parsimony reconstruction of structural and functional characters assuming an ancestral ligand-binding, ligand-dependent transcriptional activator. B. Maximum parsimony reconstruction assuming an ancestral ligand-independent activator. In both panels, ligand-regulated transcriptional activators are shown in green, with ligands in parentheses. Red, activators with no known ligand. Underlined, receptors with transcriptional activity in the absence of ligand or other modifications. Black, repressors that do not activate transcription. The ancestral NR (AncNR, green circle) is shown, with the most parsimonious character reconstruction at that node. Hash marks on branches show gains of ligand-independent activity with or without loss of ligand binding (filled and empty red boxes, respectively). States of protein structural characters are shown in the table. Specific characters are described in Fig. 5 of the main text of the paper.
