# Supplementary material for: Protein Evolution by Molecular Tinkering: Diversification of the Nuclear Receptor Superfamily from a Ligand-Dependent Ancestor
Source: PLoS Biol. 2010 Oct 5;8(10):e1000497. doi: 10.1371/journal.pbio.1000497 (PMC2950128; doi:10.1371/journal.pbio.1000497)
Supplement: Table S1 — Species, taxonomic classification, abbreviations, and accession numbers of NR sequences used in phylogenetic analyses. (2.00 MB PDF) [file pbio.1000497.s010.pdf]

Table S1. Species, taxonomy, receptor abbreviations, and accession numbers of the nuclear receptor sequences used in the phylogenetic analyses.

| Species Name | Common Name | Phylum | Lineage/Superphylum | Abbreviation | Accession # | Database |
|---|---|---|---|---|---|---|
| *Acropora millepora* | coral | Anthozoa | Cnidaria | AcrMilNR2 | Q95WG3 | UniPROTKB/TrEMBL |
| | | | | AcrMilNR6 | Q95WF8 | UniPROTKB/TrEMBL |
| | | | | AcrMilNR4 | Q95WG1 | UniPROTKB/TrEMBL |
| | | | | AcrMilNR7 | Q95WF7 | UniPROTKB/TrEMBL |
| | | | | AcrMilNR8 | Q95WF6 | UniPROTKB/TrEMBL |
| | | | | AcrMilTLL | Q95WG4 | UniPROTKB/TrEMBL |
| *Amblyomma americanum* | tick | Arthropoda | Ecdysozoa | AmbAmeEcR | O44337 | UniPROTKB/TrEMBL |
| | | | | AmbAmeRXR | O61448 | UniPROTKB/TrEMBL |
| *Amphimedon queenslandica* | sponge | Demospongia | Porifera | AmpQueNR1 | ACA04755 | Genbank |
| | | | | AmpQueNR2 | GU811658 | Genbank |
| *Aplysia californica* | sea slug | Mollusca | Lophotrochozoa | AplCalER | Q6VU64 | UniPROTKB/TrEMBL |
| *Biomphalaria glabrata* | blood fluke | Mollusca | Lophotrochozoa | BioGlaRXR | Q8T5C6 | UniPROTKB/TrEMBL |
| *Bombyx mori* | silkworm | Arthropoda | Ecdysozoa | BomMorGRF | Q9Y1J8 | UniPROTKB/TrEMBL |
| *Branchiostoma floridae* | amphioxus | Cephalochordata | Cephalochordata | BraFloCP | Q86GV5 | UniPROTKB/TrEMBL |
| | | | | BraFloNR1 | Brafl1:174225 | JGI |
| | | | | BraFloNR2 | Brafl1:124680 | JGI |
| | | | | BraFloNR3 | Brafl1:124681 | JGI |
| | | | | BraFloNR4 | Brafl1:128090 | JGI |
| | | | | BraFloNR5 | Brafl1:67032 | JGI |
| | | | | BraFloERR | Q5XTR0 | UniPROTKB/TrEMBL |
| | | | | BraFloNR6 | Brafl1:222287 | JGI |
| | | | | BraFloNR7 | Brafl1:84128 | JGI |
| | | | | BraFloNR8 | Brafl1:174611 | JGI |
| | | | | BraFloRAR | Q8MX80 | UniPROTKB/TrEMBL |
| | | | | BraFlorER | EU371730 | Genbank |
| | | | | BraFloRXR | Q8MX78 | UniPROTKB/TrEMBL |
| | | | | BraFloSR | EU371729 | Genbank |
| | | | | BraFloTH | A7L5U9 | UniPROTKB/TrEMBL |
| | | | | BraFloTR24 | Q8MX79 | UniPROTKB/TrEMBL |
| *Capitella capitata* | bristle worm | Polychaeta | Lophotrochozoa | CapCapNR1 | Capca1:219555 | JGI |
| | | | | CapCapNR2 | Capca1:222512 | JGI |
| | | | | CapCapNR3 | Capca1:226941 | JGI |
| | | | | CapCapNR4 | Capca1:227484 | JGI |
| | | | | CapCapNR5 | Capca1:227950 | JGI |
| | | | | CapCapNR6 | Capca1:62897 | JGI |
| | | | | CapCapNR7 | Capca1:224945 | JGI |
| | | | | CapCapNR8 | Capca1:168520 | JGI |
| | | | | CapCapNR9 | Capca1:167148 | JGI |
| | | | | CapCapNR10 | Capca1:93472 | JGI |
| *Carcinus maenas* | green crab | Crustacea | Ecdysozoa | CarMaeEcR | Q6RIB4 | UniPROTKB/TrEMBL |
| *Celuca pugilator* | atlantic fiddler crab | Crustacea | Ecdysozoa | CelPugEcR | O76246 | UniPROTKB/TrEMBL |
| | | | | CelPugRXR | O76241 | UniPROTKB/TrEMBL |
| *Ciona intestinalis* | transparent sea squirt | Ascidiacea | Urochordata | CioIntNR1 | ci0100142690 | JGI |
| | | | | CioIntNR2 | ci0100150442 | JGI |
| | | | | CioIntNR3 | ci0100139008 | JGI |
| | | | | CioIntNR4 | ci0100145000 | JGI |
| | | | | CioIntNR5 | ci0100146308 | JGI |
| | | | | CioIntNR6 | grail.125.34.1 | JGI |
| | | | | CioIntNR7 | grail.343.2.1 | JGI |
| | | | | CioIntNR8 | ci0100153345 | JGI |
| | | | | CioIntNR9 | ci0100143297 | JGI |
| | | | | CioIntNR10 | ci0100135919 | JGI |
| | | | | CioIntNR11 | ci0100150561 | JGI |
| | | | | CioIntThR | Q4H2P4 | UniPROTKB/TrEMBL |
| | | | | CioIntTR2/4 | Q1RPY8 | UniPROTKB/TrEMBL |
| | | | | CioIntNR12 | ci0100135732 | JGI |
| | | | | CioIntNR13 | ci0100152224 | JGI |
| | | | | CioIntNR14 | ci0100147513 | JGI |
| *Drosophila melanogaster* | fruit fly | Arthropoda | Ecdysozoa | DroMelDSF | O96680 | UniPROTKB/TrEMBL |
| | | | | DroMelE75 | P17671 | UniPROTKB/TrEMBL |
| | | | | DroMelE78 | P45447 | UniPROTKB/TrEMBL |
| | | | | DroMelEcR | P34021 | UniPROTKB/TrEMBL |
| | | | | DroMelERR | Q9VSE9 | UniPROTKB/TrEMBL |
| | | | | DroMelFAX1 | Q9VI12 | UniPROTKB/TrEMBL |
| | | | | DroMelFTZF1 | P33244 | UniPROTKB/TrEMBL |
| | | | | DroMelGRF/HR4 | Q9W539 | UniPROTKB/TrEMBL |
| | | | | DroMelHNF4 | P49866 | UniPROTKB/TrEMBL |
| | | | | DroMelHR3 | P31396 | UniPROTKB/TrEMBL |
| | | | | DroMelHR38 | P49869 | UniPROTKB/TrEMBL |
| | | | | DroMelHR39 | Q05192 | UniPROTKB/TrEMBL |
| | | | | DroMelHR78 | Q24142 | UniPROTKB/TrEMBL |
| | | | | DroMelHR96 | Q24143 | UniPROTKB/TrEMBL |
| | | | | DroMelPNR/HR51 | A1ZA01 | UniPROTKB/TrEMBL |
| | | | | DroMelSVP | Q8MRP3 | UniPROTKB/TrEMBL |
| | | | | DroMelTLL | P18102 | UniPROTKB/TrEMBL |
| | | | | DroMelUSP | P20153 | UniPROTKB/TrEMBL |
| *Fugu rubripes* | Japanese pufferfish | Euteleostomi | Vertebrata | FugRubNR1 | SINFRUP00000051057 | GTD; Genomic threading Database, http://bioinf.cs.ucl.ac.uk/GTD/ |
| | | | | FugRubNR2 | SINFRUP00000052715 | GTD; Genomic threading Database, http://bioinf.cs.ucl.ac.uk/GTD/ |
| | | | | FugRubNR3 | SINFRUP00000054267 | GTD; Genomic threading Database, http://bioinf.cs.ucl.ac.uk/GTD/ |
| | | | | FugRubNR4 | SINFRUP00000057192 | GTD; Genomic threading Database, http://bioinf.cs.ucl.ac.uk/GTD/ |
| | | | | FugRubNR5 | SINFRUP00000057832 | GTD; Genomic threading Database, http://bioinf.cs.ucl.ac.uk/GTD/ |
| | | | | FugRubNR6 | SINFRUP00000058046 | GTD; Genomic threading Database, http://bioinf.cs.ucl.ac.uk/GTD/ |
| | | | | FugRubNR7 | SINFRUP00000059292 | GTD; Genomic threading Database, http://bioinf.cs.ucl.ac.uk/GTD/ |
| | | | | FugRubNR8 | SINFRUP00000059880 | GTD; Genomic threading Database, http://bioinf.cs.ucl.ac.uk/GTD/ |
| | | | | FugRubNR9 | SINFRUP00000061614 - 15 | GTD; Genomic threading Database, http://bioinf.cs.ucl.ac.uk/GTD/ |
| | | | | FugRubNR10 | SINFRUP00000062437 | GTD; Genomic threading Database, http://bioinf.cs.ucl.ac.uk/GTD/ |
| | | | | FugRubNR11 | SINFRUP00000062708 | GTD; Genomic threading Database, http://bioinf.cs.ucl.ac.uk/GTD/ |
| | | | | FugRubNR12 | SINFRUP00000063957 | GTD; Genomic threading Database, http://bioinf.cs.ucl.ac.uk/GTD/ |
| | | | | FugRubNR13 | SINFRUP00000064369 | GTD; Genomic threading Database, http://bioinf.cs.ucl.ac.uk/GTD/ |
| | | | | FugRubNR14 | SINFRUP00000064603 | GTD; Genomic threading Database, http://bioinf.cs.ucl.ac.uk/GTD/ |
| | | | | FugRubNR15 | SINFRUP0000006485 | GTD; Genomic threading Database, http://bioinf.cs.ucl.ac.uk/GTD/ |
| | | | | FugRubNR16 | SINFRUP00000065625;<br>SINFRUP00000050176 | GTD; Genomic threading Database, http://bioinf.cs.ucl.ac.uk/GTD/ |
| | | | | FugRubNR17 | SINFRUP00000067205 | GTD; Genomic threading Database, http://bioinf.cs.ucl.ac.uk/GTD/ |
| | | | | FugRubNR18 | SINFRUP00000069536 | GTD; Genomic threading Database, http://bioinf.cs.ucl.ac.uk/GTD/ |
| | | | | FugRubNR19 | SINFRUP00000069756 | GTD; Genomic threading Database, http://bioinf.cs.ucl.ac.uk/GTD/ |
| | | | | FugRubNR20 | SINFRUP00000070604 | GTD; Genomic threading Database, http://bioinf.cs.ucl.ac.uk/GTD/ |
| | | | | FugRubNR21 | SINFRUP00000070767 | GTD; Genomic threading Database, http://bioinf.cs.ucl.ac.uk/GTD/ |
| | | | | FugRubNR22 | SINFRUP00000070795 | GTD; Genomic threading Database, http://bioinf.cs.ucl.ac.uk/GTD/ |
| | | | | FugRubNR23 | SINFRUP00000071164 | GTD; Genomic threading Database, http://bioinf.cs.ucl.ac.uk/GTD/ |
| | | | | FugRubNR24 | SINFRUP00000071695 | GTD; Genomic threading Database, http://bioinf.cs.ucl.ac.uk/GTD/ |
| | | | | FugRubNR25 | SINFRUP00000072134 | GTD; Genomic threading Database, http://bioinf.cs.ucl.ac.uk/GTD/ |
| | | | | FugRubNR26 | SINFRUP00000072315 | GTD; Genomic threading Database, http://bioinf.cs.ucl.ac.uk/GTD/ |
| | | | | FugRubNR27 | SINFRUP00000073157 | GTD; Genomic threading Database, http://bioinf.cs.ucl.ac.uk/GTD/ |
| | | | | FugRubNR28 | SINFRUP00000074502 | GTD; Genomic threading Database, http://bioinf.cs.ucl.ac.uk/GTD/ |
| | | | | FugRubNR29 | SINFRUP00000074673 | GTD; Genomic threading Database, http://bioinf.cs.ucl.ac.uk/GTD/ |
| | | | | FugRubNR30 | SINFRUP00000076812 | GTD; Genomic threading Database, http://bioinf.cs.ucl.ac.uk/GTD/ |
| | | | | FugRubNR31 | SINFRUP00000077845 | GTD; Genomic threading Database, http://bioinf.cs.ucl.ac.uk/GTD/ |
| | | | | FugRubNR32 | SINFRUP00000079088 | GTD; Genomic threading Database, http://bioinf.cs.ucl.ac.uk/GTD/ |
| | | | | FugRubNR33 | SINFRUP00000079602 | GTD; Genomic threading Database, http://bioinf.cs.ucl.ac.uk/GTD/ |
| | | | | FugRubNR34 | SINFRUP00000080805 | GTD; Genomic threading Database, http://bioinf.cs.ucl.ac.uk/GTD/ |
| | | | | FugRubNR35 | SINFRUP00000080916 | GTD; Genomic threading Database, http://bioinf.cs.ucl.ac.uk/GTD/ |
| | | | | FugRubNR36 | SINFRUP00000081538 | GTD; Genomic threading Database, http://bioinf.cs.ucl.ac.uk/GTD/ |
| | | | | FugRubNR37 | SINFRUP00000082389 | GTD; Genomic threading Database, http://bioinf.cs.ucl.ac.uk/GTD/ |
| | | | | FugRubNR38 | SINFRUP00000085838 | GTD; Genomic threading Database, http://bioinf.cs.ucl.ac.uk/GTD/ |
| | | | | FugRubNR39 | SINFRUP00000090296 | GTD; Genomic threading Database, http://bioinf.cs.ucl.ac.uk/GTD/ |
| | | | | FugRubNR40 | SINFRUP00000091008 | GTD; Genomic threading Database, http://bioinf.cs.ucl.ac.uk/GTD/ |
| | | | | FugRubNR41 | SINFRUP00000079067 | GTD; Genomic threading Database, http://bioinf.cs.ucl.ac.uk/GTD/ |
| | | | | FugRubNR42 | SINFRUP0000008748 | GTD; Genomic threading Database, http://bioinf.cs.ucl.ac.uk/GTD/ |
| | | | | FugRubNR43 | SINFRUP00000054948 | GTD; Genomic threading Database, http://bioinf.cs.ucl.ac.uk/GTD/ |

| | | | | | | |
|---|---|---|---|---|---|---|
| *Homo sapiens* | human | Vertebrata | Chordata | HomSapAR | P10275 | UniPROTKB/TrEMBL |
| | | | | HomSapCAR | Q14994 | UniPROTKB/TrEMBL |
| | | | | HomSapCP1 | P10589 | UniPROTKB/TrEMBL |
| | | | | HomSapCP2 | P24468 | UniPROTKB/TrEMBL |
| | | | | HomSapEAR2 | P10588 | UniPROTKB/TrEMBL |
| | | | | HomSapERa | P03372 | UniPROTKB/TrEMBL |
| | | | | HomSapERb | Q9UHD3 | UniPROTKB/TrEMBL |
| | | | | HomSapERRa | P11474 | UniPROTKB/TrEMBL |
| | | | | HomSapERRb | O95718 | UniPROTKB/TrEMBL |
| | | | | HomSapERRg | AAQ93376 | UniPROTKB/TrEMBL |
| | | | | HomSapFTZF1/LRH-1 | Q9UEC0 | UniPROTKB/TrEMBL |
| | | | | HomSapFXR | Q96RI1 | UniPROTKB/TrEMBL |
| | | | | HomSapGCNF | Q15406 | UniPROTKB/TrEMBL |
| | | | | HomSapGR | P04150 | UniPROTKB/TrEMBL |
| | | | | HomSapHMR | P22736 | UniPROTKB/TrEMBL |
| | | | | HomSapHNF4a | Q9NQH0 | UniPROTKB/TrEMBL |
| | | | | HomSapHNF4g | Q14541 | UniPROTKB/TrEMBL |
| | | | | HomSapLXRa | Q13133 | UniPROTKB/TrEMBL |
| | | | | HomSapLXRb | P55055 | UniPROTKB/TrEMBL |
| | | | | HomSapMR | P08235 | UniPROTKB/TrEMBL |
| | | | | HomSapNOR1 | Q92570 | UniPROTKB/TrEMBL |
| | | | | HomSapNurr1 | P43354 | UniPROTKB/TrEMBL |
| | | | | HomSapPNR | Q9Y5X4 | UniPROTKB/TrEMBL |
| | | | | HomSapPPARa | Q07869 | UniPROTKB/TrEMBL |
| | | | | HomSapPPARd | Q03181 | UniPROTKB/TrEMBL |
| | | | | HomSapPPARg | Q96J12 | UniPROTKB/TrEMBL |
| | | | | HomSapPR | P06401 | UniPROTKB/TrEMBL |
| | | | | HomSapPXR | O75469 | UniPROTKB/TrEMBL |
| | | | | HomSapRARa | P10276 | UniPROTKB/TrEMBL |
| | | | | HomSapRARb | P10826 | UniPROTKB/TrEMBL |
| | | | | HomSapRARg | P13631 | UniPROTKB/TrEMBL |
| | | | | HomSapRevERBa | P20393 | UniPROTKB/TrEMBL |
| | | | | HomSapRevERBb | Q14995 | UniPROTKB/TrEMBL |
| | | | | HomSapRORa | P35398 | UniPROTKB/TrEMBL |
| | | | | HomSapRORb | Q92753 | UniPROTKB/TrEMBL |
| | | | | HomSapRORg | P51449 | UniPROTKB/TrEMBL |
| | | | | HomSapRXRa | P19793 | UniPROTKB/TrEMBL |
| | | | | HomSapRXRb | P28702 | UniPROTKB/TrEMBL |
| | | | | HomSapRXRg | P48443 | UniPROTKB/TrEMBL |
| | | | | HomSapSF1 | Q13285 | UniPROTKB/TrEMBL |
| | | | | HomSapThRa1 | P10827 | UniPROTKB/TrEMBL |
| | | | | HomSapThrb1 | P10828 | UniPROTKB/TrEMBL |
| | | | | HomSapTLX | Q9Y466 | UniPROTKB/TrEMBL |
| | | | | HomSapTR2 | P13056 | UniPROTKB/TrEMBL |
| | | | | HomSapTR4 | P49116 | UniPROTKB/TrEMBL |
| | | | | HomSapVDR | P11473 | UniPROTKB/TrEMBL |
| *Hydra vulgaris* | hydra | Hydrozoa | Cnidaria | HydVulCP | Q66MI8 | UniPROTKB/TrEMBL |
| *Latimeria chalumnae* | coelocanth | Euteleostomi | Vertebrata | LatChaCP | Q6EEB7 | UniPROTKB/TrEMBL |
| *Leucoraja erinacea* | little skate | Elasmobranchii | Vertebrata | LeuEriFXR | A5HEI3 | UniPROTKB/TrEMBL |
| *Lithobius forficatus* | brown centipede | Annelida | Lophotrochozoa | LitForUSPRXR | Q86LV1 | UniPROTKB/TrEMBL |
| *Lottia gigantea* | limpet | Gastropoda | Lophotrochozoa | LotGigNR1 | LgGsHFWreduced.10352 | JGI |
| | | | | LotGigNR2 | LgGsHFWreduced.1060 | JGI |
| | | | | LotGigNR3 | LgGsHFWreduced.6539 | JGI |
| | | | | LotGigNR4 | LgGsHFWreduced.2044 | JGI |
| | | | | LotGigNR5 | LgGsHFWreduced.3996 | JGI |
| | | | | LotGigNR6 | Lotgi1:64143 | JGI |
| | | | | LotGigNR7 | Lotgi1:122023 | JGI |
| | | | | LotGigNR8 | LgGsHFWreduced.11616 | JGI |
| | | | | LotGigNR9 | Lotgi1:132166 | JGI |
| | | | | LotGigNR10 | Lotgi1:72980 | JGI |
| | | | | LotGigNR11 | Lotgi1:162299 | JGI |
| | | | | LotGigNR12 | LgGsHFWreduced.1498 | JGI |
| | | | | LotGigNR13 | LgGsHFWreduced.915 | JGI |
| | | | | LotGigNR14 | Lotgi1:207303 | JGI |
| | | | | LotGigNR15 | Lotgi1:180136 | JGI |
| | | | | LotGigNR16 | Lotgi1:174409 | JGI |
| | | | | LotGigNR17 | Lotgi1:211160 | JGI |
| | | | | LotGigNR18 | LgGsHFWreduced.1922 | JGI |
| | | | | LotGigNR19 | Lotgi1:200640 | JGI |
| | | | | LotGigNR20 | Lotgi1:136477 | JGI |
| | | | | LotGigNR21 | Lotgi1:29003 | JGI |
| | | | | LotGigNR22 | LgGsHFWreduced.11289 | JGI |
| | | | | LotGigNR23 | LgGsHFWreduced.693 | JGI |
| | | | | LotGigNR24 | LgGsHFWreduced.446 | JGI |
| | | | | LotGigNR25 | Lotgi1:130367 | JGI |
| | | | | LotGigNR26 | Lotgi1:160372 | JGI |
| *Lymnaea stagnalis* | great pond snail | Gastropoda | Lophotrochozoa | LymStaRXR | Q5I7G2 | UniPROTKB/TrEMBL |
| *Marisa cornuarietis* | apple snail | Gastropoda | Lophotrochozoa | MarCorERR | Q06AL0 | UniPROTKB/TrEMBL |
| *Metapenaeus ensis* | shrimp | Crustacea | Ecdysozoa | MetEnsE75 | Q9Y0D1 | UniPROTKB/TrEMBL |
| | | | | MetEnsFTZF1 | O77245 | UniPROTKB/TrEMBL |
| *Myxine glutinosa* | Atlantic hagfish | Craniata | Vertebrata | MyxGluCR | Q1KXY6 | UniPROTKB/TrEMBL |
| | | | | MyxGluSR2 | Q1KXY5 | UniPROTKB/TrEMBL |
| *Nematostella vectensis* | starlet sea anemone | Anthozoa | Cnidaria | NemVecNR1 | Nemve1:203423 | JGI |
| | | | | NemVecNR2 | Nemve1:242271 | JGI |
| | | | | NemVecNR3 | Nemve1:165424 | JGI |
| | | | | NemVecNR4 | Nemve1:189134 | JGI |
| | | | | NemVecNR5 | Nemve1:89471 | JGI |
| | | | | NemVecNR6 | Nemve1:108851 | JGI |
| | | | | NemVecNR7 | Nemve1:101676 | JGI |
| | | | | NemVecNR8 | :Nemve1:108851 | JGI |
| | | | | NemVecNR9 | Nemve1:93844 | JGI |
| | | | | NemVecNR10 | Nemve1:134436 | JGI |
| | | | | NemVecNR11 | Nemve1:183874 | JGI |
| | | | | NemVecNR12 | Nemve1:99425 | JGI |
| | | | | NemVecNR13 | Nemve1:169225 | JGI |
| | | | | NemVecNR14 | Nemve1:114090 | JGI |
| | | | | NemVecNR15 | Nemve1:132075 | JGI |
| | | | | NemVecNR16 | Nemve1:167880 | JGI |
| | | | | NemVecNR17 | Nemve1:209681 | JGI |
| *Nucella lapillus* | Atlantic dogwinkle | Gastropoda | Lophotrochozoa | NucLapER | A5YVJ5 | UniPROTKB/TrEMBL |
| | | | | NucLapRXR | A7LIS4 | UniPROTKB/TrEMBL |
| *Octopus vulgaris* | octopus | Cephalopoda | Mollusca | OctVulER | Q19AB0 | UniPROTKB/TrEMBL |
| *Oikopleura dioicia* | tunicate | | Urochordata | OikDipRXR | A7J002 | UniPROTKB/TrEMBL |
| *Paranemertes peregrina* | purple ribbon worm | Nemertea | Lophotrochozoa | ParPerERR | HM745933 | Genbank |
| *Penaeus japonicus* | Kuruma prawn | Crustacea | Ecdysozoa | PenJapRXR | A7BJ54 | UniPROTKB/TrEMBL |
| *Petromyzon marinus* | sea lamprey | Hyperoartia | Vertebrata | PetMarCP | O42509 | UniPROTKB/TrEMBL |
| | | | | PetMarCR | Q90ZM7 | UniPROTKB/TrEMBL |
| | | | | PetMarER | Q90ZM8 | UniPROTKB/TrEMBL |
| | | | | PetMarPR | Q90ZM6 | UniPROTKB/TrEMBL |
| | | | | PetMarRAR | Q52WZ5 | UniPROTKB/TrEMBL |
| | | | | PetMarRXR | Q90Y01 | UniPROTKB/TrEMBL |
| | | | | PetMarRXR2 | Q2PK06 | UniPROTKB/TrEMBL |
| | | | | PetMarTH | Q2PK09 | UniPROTKB/TrEMBL |
| | | | | PetMarTH2 | Q2PK08 | UniPROTKB/TrEMBL |
| | | | | PetMarVDR | Q7ZZY9 | UniPROTKB/TrEMBL |

| | | | | | | |
|---|---|---|---|---|---|---|
| *Platynereis dumerilli* | polychaete worm | Annelida | Lophotrochozoa | PlaDumER | C0IR13 | UniPROTKB/TrEMBL |
| *Pocillopora damicornis* | bird's nest coral | Anthozoa | Cnidaria | PocDamNR6 | A7L8D5 | UniPROTKB/TrEMBL |
| *Polyandrocarpa misakiensis* | | Ascidiacea | Urochordata | PolMisRAR | P91779 | UniPROTKB/TrEMBL |
| | | | | PolMisRXR | Q9UAF1 | UniPROTKB/TrEMBL |
| *Saccoglossus kowalevskii* | acorn worm | | Hemichordata | SacKowTLL | Q7YTB9 | UniPROTKB/TrEMBL |
| *Schistosoma mansoni* | | | | SchManTR24 | Q4KXQ1 | UniPROTKB/TrEMBL |
| | | | | SchManFTZF | Q9BPL0 | UniPROTKB/TrEMBL |
| *Strongylocentrotus purpuratus* | purple sea urchin | Echinoidea | Echinodermata | StrPurNR1 | UPI0000584A1E | NCBI |
| | | | | StrPurNR2 | XP_782295.1 | NCBI |
| | | | | StrPurNR3 | XP_785820.1 | NCBI |
| | | | | StrPurNR4 | XP_784483 | NCBI |
| | | | | StrPurNR5 | XP_791919.1 | NCBI |
| | | | | StrPurNR6 | UPI0000586F42 | SMART |
| | | | | StrPurNR7 | UPI0000E469E4 | SMART |
| | | | | StrPurNR8 | NP_001020384.1 | NCBI |
| | | | | StrPurNR9 | XP_780389.1 | NCBI |
| | | | | StrPurNR10 | XP_779997.1 | NCBI |
| | | | | StrPurNR11 | UPI0000E4819E | SMART |
| | | | | StrPurNR12 | XP_786266.1 | NCBI |
| | | | | StrPurNR13 | XP_795547.1 | NCBI |
| | | | | StrPurNR14 | XP_780706.1 | NCBI |
| | | | | StrPurNR15 | XP_795547 | NCBI |
| | | | | StrPurNR16 | XP_781750.1 | NCBI |
| | | | | StrPurNR17 | XP_784429.2 | NCBI |
| | | | | StrPurNR18 | XP_779976.1 | NCBI |
| | | | | StrPurNR19 | XP_784691.1 | NCBI |
| | | | | StrPurNR20 | XP_001201896 | NCBI |
| | | | | StrPurNR21 | XP_792757.1 | NCBI |
| | | | | StrPurNR22 | XP_789465.1 | NCBI |
| | | | | StrPurNR23 | XP_782884.1 | NCBI |
| | | | | StrPurNR24 | UPI0000E4834A | SMART |
| *Suberites domuncula* | sponge | Demospongia | Porifera | SubDomRXR | Q8I748 | UniPROTKB/TrEMBL |
| *Scyliorhinus canicula* | smaller spotted catshark | Elasmobranch | Vertebrata | SycCanCP | AAS49607 | Genbank |
| | | | | SycCanTH | ABS11251 | Genbank |
| *Thais clavigera* | whelk | Gastropoda | Mollusca | ThaClaER | Q86B27 | UniPROTKB/TrEMBL |
| | | | | ThaClaRXR | Q66TQ0 | UniPROTKB/TrEMBL |
| *Trichoplax adhaerens* | | | Placozoa | TriAdhCP | Triad1:21656 | JGI |
| | | | | TriAdhERR | Triad1:16711 | JGI |
| | | | | TriAdhHNF4 | Triad1:50786 | JGI |
| | | | | TriAdhRXR | Triad1:49897 | JGI |
| *Tripedalia cystophora* | box jelly | Cubozoa | Cnidaria | TriCysRXR | O96562 | UniPROTKB/TrEMBL |
