# Supplementary material for: Protein Evolution by Molecular Tinkering: Diversification of the Nuclear Receptor Superfamily from a Ligand-Dependent Ancestor
Source: PLoS Biol. 2010 Oct 5;8(10):e1000497. doi: 10.1371/journal.pbio.1000497 (PMC2950128; doi:10.1371/journal.pbio.1000497)
Supplement: Table S2 — Mutations known to cause constitutive activity in liganded NRs or ligand-activation in constitutive receptors. (0.18 MB PDF) [file pbio.1000497.s011.pdf]

Receptor	Mutation	Effect	Citation
Human ERα	A350F	Constitutive activity	Chen et al., J. Biol. Chem. 276:28645, 2001
Human ERα	E380Q	Constitutive activity	Lazennec G, et al., Mol. Endo. 11:1375, 1997
Human ERα	L536A	Constitutive activity	Zhao et al., J. Biol. Chem. 278:27278, 2003
Human ERα	L536E	Constitutive activity	Zhao et al., J. Biol. Chem. 278:27278, 2003
Human ERα	L536G	Constitutive activity	Zhao et al., J. Biol. Chem. 278:27278, 2003
Human ERα	L536K	Constitutive activity	Zhao et al., J. Biol. Chem. 278:27278, 2003
Human ERα	L536N	Constitutive activity	Zhao et al., J. Biol. Chem. 278:27278, 2003
Human ERα	Y537A	Constitutive activity	Weis et al., Mol. Endo. 10:1388, 1996
Human ERα	Y537N	Constitutive activity	Zhong and Skafar, Biochemistry 41:4209, 2002
Human ERα	Y537S	Constitutive activity	Weis et al., Mol. Endo. 10:1388, 1996
Human ERα	Y541A	Constitutive activity	White et al., EMBO J., 16:1427, 1997
Human ERα	Y541D	Constitutive activity	White et al., EMBO J., 16:1427, 1997
Human ERα	Y541E	Constitutive activity	White et al., EMBO J., 16:1427, 1997
Human PR	M759A	Constitutive activity	Zhang et al., PNAS 102:2707, 2005
Human PR	M759L	Constitutive activity	Zhang et al., PNAS 102:2707, 2005
Human PXR	H407A	Constitutive activity	Ostberg et al., Eur. J. Biochem. 269:4896, 2002
Human RARα	A392R	Constitutive activity	Benko et al., J. Biol. Chem. 278:43797, 2003
Mouse RXRα	F318A	Constitutive activity	Vivat et al., EMBO J 16:5697, 1997
Mouse RXRα	F318L	Constitutive activity	Vivat et al., EMBO J 16:5697, 1997
Mouse RXRα	F318V	Constitutive activity	Vivat et al., EMBO J 16:5697, 1997
Human ERRα	F329A	Reduced constitutive activity; receptor becomes ligand-activated by antagonists of wild-type receptor, but at levels below that of wild-type baseline	Chen et al., J. Biol. Chem. 276:28645, 2001

Table S2. Nuclear receptor mutations that cause constitutive activity in liganded receptors or ligand-activation in constitutive receptors without activating ligands. Mutations were identified by querying the Nuclear Receptors Mutations Database as www.receptors.org and by literature searches and verified by consulting the primary publication.
