# Supplementary material for: Protein Evolution by Molecular Tinkering: Diversification of the Nuclear Receptor Superfamily from a Ligand-Dependent Ancestor
Source: PLoS Biol. 2010 Oct 5;8(10):e1000497. doi: 10.1371/journal.pbio.1000497 (PMC2950128; doi:10.1371/journal.pbio.1000497)
Supplement: Table S3 — Sites in NR alignment classified by evolutionary rate. (1.10 MB PDF) [file pbio.1000497.s012.pdf]

Table S3. Sites in NR alignment classified by evolutionary rate.

Posterior probabilities for site classes, by site							n=64	n=49
**Site Number**	**1**	**2**	**3**	**4**	**5**	**6**	**7**	**8**
**Rate**	0.07	0.21	0.38	0.58	0.83	1.17	1.69	3.07
**1**	1.00	0.00	0.00	0.00	0.00	0.00	0.00	0.00
**2**	0.00	0.00	0.00	0.00	0.01	0.94	0.05	0.00
**3**	0.00	0.96	0.04	0.00	0.00	0.00	0.00	0.00
**4**	1.00	0.00	0.00	0.00	0.00	0.00	0.00	0.00
**5**	0.00	0.00	0.00	0.17	0.81	0.03	0.00	0.00
**6**	0.98	0.02	0.00	0.00	0.00	0.00	0.00	0.00
**7**	0.00	0.00	0.00	0.00	0.45	0.55	0.00	0.00
**8**	0.00	1.00	0.00	0.00	0.00	0.00	0.00	0.00
**9**	0.00	0.99	0.01	0.00	0.00	0.00	0.00	0.00
**10**	0.84	0.16	0.00	0.00	0.00	0.00	0.00	0.00
**11**	0.00	0.00	0.00	0.10	0.86	0.05	0.00	0.00
**12**	1.00	0.00	0.00	0.00	0.00	0.00	0.00	0.00
**13**	0.99	0.01	0.00	0.00	0.00	0.00	0.00	0.00
**14**	0.41	0.59	0.00	0.00	0.00	0.00	0.00	0.00
**15**	0.00	0.49	0.51	0.00	0.00	0.00	0.00	0.00
**16**	0.00	0.00	0.00	0.00	0.05	0.95	0.01	0.00
**17**	0.00	0.01	0.98	0.01	0.00	0.00	0.00	0.00
**18**	1.00	0.00	0.00	0.00	0.00	0.00	0.00	0.00
**19**	1.00	0.00	0.00	0.00	0.00	0.00	0.00	0.00
**20**	0.47	0.53	0.00	0.00	0.00	0.00	0.00	0.00
**21**	1.00	0.00	0.00	0.00	0.00	0.00	0.00	0.00
**22**	0.99	0.01	0.00	0.00	0.00	0.00	0.00	0.00
**23**	0.01	0.96	0.03	0.00	0.00	0.00	0.00	0.00
**24**	1.00	0.00	0.00	0.00	0.00	0.00	0.00	0.00
**25**	1.00	0.00	0.00	0.00	0.00	0.00	0.00	0.00
**26**	0.88	0.12	0.00	0.00	0.00	0.00	0.00	0.00
**27**	1.00	0.00	0.00	0.00	0.00	0.00	0.00	0.00
**28**	0.00	0.98	0.02	0.00	0.00	0.00	0.00	0.00
**29**	0.00	0.00	0.04	0.94	0.02	0.00	0.00	0.00
**30**	0.00	0.01	0.93	0.06	0.00	0.00	0.00	0.00
**31**	0.00	0.00	0.00	0.29	0.70	0.00	0.00	0.00
**32**	0.00	0.00	0.00	0.00	0.00	0.98	0.02	0.00
**33**	0.00	0.00	0.00	0.01	0.77	0.22	0.00	0.00
**34**	0.14	0.14	0.13	0.13	0.13	0.12	0.11	0.10
**35**	0.13	0.13	0.13	0.13	0.13	0.12	0.12	0.11
**36**	0.00	0.00	0.00	0.00	0.00	0.00	0.01	0.99
**37**	0.00	0.00	0.08	0.79	0.13	0.00	0.00	0.00
**38**	0.00	0.00	0.00	0.00	0.00	0.08	0.92	0.00
**39**	1.00	0.00	0.00	0.00	0.00	0.00	0.00	0.00
**40**	0.00	0.00	0.00	0.00	0.11	0.89	0.00	0.00
**41**	0.00	0.00	0.00	0.00	0.00	0.25	0.75	0.00
**42**	0.00	0.00	0.00	0.00	0.00	0.00	1.00	0.00
**43**	0.00	0.00	0.02	0.09	0.23	0.32	0.25	0.10
**44**	0.00	0.00	0.01	0.03	0.07	0.16	0.29	0.44
**45**	0.00	0.00	0.00	0.00	0.02	0.07	0.23	0.67
**46**	0.00	0.00	0.00	0.00	0.03	0.40	0.57	0.00
**47**	0.00	0.00	0.00	0.00	0.01	0.95	0.04	0.00
**48**	0.00	0.00	0.00	0.00	0.00	0.00	1.00	0.00
**49**	1.00	0.00	0.00	0.00	0.00	0.00	0.00	0.00
**50**	0.00	0.00	0.00	0.00	0.00	0.00	0.01	0.99
**51**	0.00	0.00	0.17	0.83	0.00	0.00	0.00	0.00
**52**	0.00	0.00	0.01	0.88	0.11	0.00	0.00	0.00
**53**	0.00	0.00	0.08	0.89	0.03	0.00	0.00	0.00
**54**	0.00	0.00	0.00	0.00	0.00	0.72	0.28	0.00
**55**	0.00	0.00	0.10	0.89	0.01	0.00	0.00	0.00
**56**	1.00	0.00	0.00	0.00	0.00	0.00	0.00	0.00
**57**	0.75	0.25	0.00	0.00	0.00	0.00	0.00	0.00
**58**	0.00	0.02	0.94	0.05	0.00	0.00	0.00	0.00
**59**	1.00	0.00	0.00	0.00	0.00	0.00	0.00	0.00
**60**	0.00	0.75	0.25	0.00	0.00	0.00	0.00	0.00
**61**	0.00	0.00	0.00	0.00	0.17	0.83	0.00	0.00
**62**	1.00	0.00	0.00	0.00	0.00	0.00	0.00	0.00
**63**	1.00	0.00	0.00	0.00	0.00	0.00	0.00	0.00
**64**	0.00	0.00	0.00	0.28	0.70	0.02	0.00	0.00
**65**	0.00	0.00	0.00	0.00	0.54	0.46	0.00	0.00
**66**	0.00	0.95	0.05	0.00	0.00	0.00	0.00	0.00
**67**	1.00	0.00	0.00	0.00	0.00	0.00	0.00	0.00
**68**	0.00	0.00	0.00	0.00	0.00	0.22	0.78	0.00
**69**	0.00	0.00	0.00	0.00	0.00	0.00	0.83	0.17
**70**	0.00	0.00	0.00	0.00	0.60	0.40	0.00	0.00
**71**	0.00	0.87	0.13	0.00	0.00	0.00	0.00	0.00
**72**	1.00	0.00	0.00	0.00	0.00	0.00	0.00	0.00
**73**	0.00	0.00	0.02	0.94	0.04	0.00	0.00	0.00
**74**	0.00	0.00	0.00	0.00	0.01	0.96	0.04	0.00
**75**	0.00	0.00	0.11	0.86	0.03	0.00	0.00	0.00
**76**	0.00	0.00	0.91	0.09	0.00	0.00	0.00	0.00
**77**	0.00	0.30	0.70	0.00	0.00	0.00	0.00	0.00
**78**	0.00	0.93	0.07	0.00	0.00	0.00	0.00	0.00
**79**	0.00	0.00	0.00	0.00	0.10	0.90	0.01	0.00
**80**	0.00	0.00	0.64	0.35	0.00	0.00	0.00	0.00
**81**	0.00	0.55	0.45	0.00	0.00	0.00	0.00	0.00
**82**	0.00	0.00	0.00	0.03	0.68	0.30	0.00	0.00
**83**	0.00	0.00	0.00	0.14	0.73	0.13	0.00	0.00
**84**	0.00	0.00	0.00	0.22	0.67	0.11	0.00	0.00
**85**	0.00	0.00	0.00	0.07	0.60	0.33	0.00	0.00
**86**	0.00	0.00	0.00	0.00	0.08	0.71	0.21	0.00
**87**	0.00	0.00	0.00	0.00	0.00	0.22	0.78	0.00
**88**	0.00	0.00	0.00	0.00	0.01	0.95	0.04	0.00

Table S3 page 2

**89**	0.00	0.00	0.00	0.00	0.00	0.35	0.65	0.00
**90**	0.00	0.00	0.00	0.00	0.00	0.00	1.00	0.00
**91**	0.00	0.00	0.00	0.00	0.15	0.85	0.00	0.00
**92**	0.00	0.00	0.00	0.04	0.94	0.02	0.00	0.00
**93**	0.00	0.00	0.00	0.00	0.00	0.01	0.99	0.00
**94**	0.00	0.00	0.00	0.00	0.06	0.94	0.00	0.00
**95**	0.00	0.00	0.02	0.84	0.15	0.00	0.00	0.00
**96**	0.00	0.00	0.00	0.00	0.00	0.02	0.98	0.00
**97**	0.00	0.00	0.00	0.00	0.00	0.00	1.00	0.00
**98**	0.00	0.00	0.00	0.00	0.02	0.97	0.01	0.00
**99**	0.00	0.00	0.36	0.64	0.00	0.00	0.00	0.00
**100**	0.00	0.00	0.00	0.00	0.00	0.03	0.97	0.00
**101**	0.00	0.08	0.71	0.21	0.00	0.00	0.00	0.00
**102**	0.00	0.00	0.17	0.82	0.00	0.00	0.00	0.00
**103**	0.00	0.01	0.88	0.11	0.00	0.00	0.00	0.00
**104**	0.00	0.00	0.00	0.00	0.00	0.00	1.00	0.00
**105**	0.00	0.00	0.00	0.00	0.52	0.48	0.00	0.00
**106**	0.00	0.00	0.00	0.52	0.47	0.00	0.00	0.00
**107**	0.00	0.00	0.00	0.00	0.40	0.60	0.00	0.00
**108**	0.00	0.75	0.24	0.00	0.00	0.00	0.00	0.00
**109**	0.00	0.00	0.00	0.00	0.00	0.00	0.00	1.00
**110**	0.00	0.00	0.00	0.00	0.00	0.00	0.00	1.00
**111**	0.00	0.01	0.71	0.28	0.00	0.00	0.00	0.00
**112**	0.00	0.00	0.00	0.00	0.00	0.00	0.98	0.02
**113**	0.00	0.00	0.00	0.00	0.00	0.00	1.00	0.00
**114**	0.00	0.00	0.00	0.00	0.00	0.00	1.00	0.00
**115**	0.00	0.99	0.01	0.00	0.00	0.00	0.00	0.00
**116**	0.07	0.93	0.00	0.00	0.00	0.00	0.00	0.00
**117**	0.00	0.00	0.00	0.06	0.93	0.01	0.00	0.00
**118**	0.00	0.00	0.00	0.00	0.00	0.00	1.00	0.00
**119**	0.00	0.75	0.25	0.00	0.00	0.00	0.00	0.00
**120**	0.00	0.00	0.24	0.74	0.02	0.00	0.00	0.00
**121**	0.00	0.00	0.30	0.69	0.01	0.00	0.00	0.00
**122**	0.00	0.00	0.00	0.00	0.00	0.05	0.95	0.00
**123**	0.00	0.00	0.00	0.00	0.13	0.87	0.01	0.00
**124**	0.00	0.00	0.00	0.01	0.52	0.47	0.00	0.00
**125**	0.00	0.00	0.00	0.00	0.61	0.39	0.00	0.00
**126**	0.00	0.00	0.77	0.23	0.00	0.00	0.00	0.00
**127**	0.00	0.00	0.00	0.00	0.38	0.62	0.00	0.00
**128**	0.00	0.00	0.00	0.01	0.75	0.24	0.00	0.00
**129**	0.00	0.00	0.00	0.00	0.00	0.00	1.00	0.00
**130**	0.00	0.00	0.00	0.06	0.89	0.05	0.00	0.00
**131**	0.00	0.00	0.00	0.00	0.21	0.79	0.00	0.00
**132**	0.00	0.00	0.00	0.00	0.00	0.36	0.64	0.00
**133**	0.00	0.00	0.00	0.00	0.01	0.97	0.02	0.00
**134**	0.00	0.00	0.00	0.00	0.00	0.15	0.85	0.00
**135**	0.00	0.00	0.00	0.00	0.02	0.95	0.03	0.00
**136**	0.00	0.00	0.00	0.00	0.46	0.54	0.00	0.00
**137**	0.00	0.00	0.00	0.00	0.00	0.00	0.12	0.88
**138**	0.00	0.00	0.00	0.00	0.00	0.00	0.00	1.00
**139**	0.00	0.00	0.00	0.00	0.00	0.06	0.94	0.00
**140**	0.00	0.00	0.00	0.00	0.00	0.00	0.18	0.82
**141**	0.00	0.00	0.00	0.00	0.00	0.01	0.99	0.00
**142**	0.00	0.00	0.00	0.00	0.00	0.00	1.00	0.00
**143**	0.00	0.00	0.00	0.00	0.00	0.00	1.00	0.00
**144**	0.00	0.00	0.00	0.00	0.00	0.13	0.87	0.00
**145**	0.00	0.00	0.00	0.00	0.00	0.00	0.00	1.00
**146**	0.00	0.00	0.00	0.00	0.00	0.00	0.00	1.00
**147**	0.00	0.00	0.00	0.00	0.00	0.00	0.55	0.45
**148**	0.00	0.00	0.00	0.00	0.00	0.00	0.99	0.01
**149**	0.00	0.00	0.00	0.00	0.00	0.00	0.05	0.95
**150**	0.00	0.00	0.00	0.00	0.00	0.00	0.00	1.00
**151**	0.00	0.00	0.00	0.00	0.00	0.00	0.00	1.00
**152**	0.00	0.00	0.00	0.00	0.00	0.00	0.49	0.51
**153**	0.00	0.00	0.00	0.00	0.00	0.00	0.00	1.00
**154**	0.00	0.00	0.00	0.00	0.00	0.00	0.00	1.00
**155**	0.00	0.00	0.00	0.00	0.00	0.00	0.00	1.00
**156**	0.00	0.00	0.00	0.00	0.00	0.00	0.00	1.00
**157**	0.00	0.00	0.00	0.00	0.00	0.00	0.00	1.00
**158**	0.00	0.00	0.00	0.00	0.00	0.00	0.27	0.73
**159**	0.00	0.00	0.00	0.00	0.00	0.00	1.00	0.00
**160**	0.00	0.00	0.00	0.00	0.00	0.00	0.09	0.91
**161**	0.00	0.03	0.10	0.19	0.25	0.25	0.16	0.02
**162**	0.00	0.02	0.07	0.17	0.26	0.28	0.18	0.02
**163**	0.00	0.00	0.02	0.07	0.18	0.30	0.33	0.09
**164**	0.00	0.00	0.00	0.00	0.00	0.00	1.00	0.00
**165**	0.00	0.00	0.00	0.00	0.00	0.00	0.21	0.79
**166**	0.00	0.00	0.00	0.00	0.01	0.95	0.04	0.00
**167**	0.00	0.00	0.00	0.00	0.00	0.06	0.94	0.00
**168**	0.00	0.00	0.00	0.00	0.00	0.00	1.00	0.00
**169**	0.00	0.00	0.00	0.00	0.28	0.72	0.00	0.00
**170**	0.00	0.00	0.00	0.00	0.00	0.11	0.89	0.00
**171**	0.00	0.00	0.00	0.00	0.00	0.00	0.00	1.00
**172**	0.00	0.00	0.00	0.00	0.00	0.04	0.96	0.00
**173**	0.00	0.00	0.00	0.00	0.00	0.32	0.68	0.00
**174**	0.00	0.00	0.00	0.00	0.00	0.00	0.98	0.02
**175**	0.00	0.00	0.00	0.00	0.00	0.00	0.00	1.00
**176**	0.00	0.00	0.00	0.00	0.00	0.72	0.28	0.00
**177**	0.00	0.00	0.00	0.00	0.00	0.00	0.00	1.00
**178**	0.00	0.00	0.00	0.00	0.00	0.19	0.81	0.00
**179**	0.00	0.00	0.00	0.04	0.93	0.03	0.00	0.00
**180**	0.00	0.00	0.00	0.00	0.00	0.00	0.12	0.88
**181**	0.00	0.00	0.00	0.00	0.00	0.49	0.51	0.00
**182**	0.00	0.93	0.07	0.00	0.00	0.00	0.00	0.00
**183**	0.00	0.00	0.00	0.00	0.00	0.00	1.00	0.00
**184**	0.00	0.00	0.00	0.01	0.91	0.09	0.00	0.00

Table S3 page 3

**185**	0.00	0.00	0.02	0.65	0.33	0.00	0.00	0.00
**186**	0.00	0.00	0.00	0.35	0.64	0.01	0.00	0.00
**187**	0.00	0.00	0.00	0.00	0.81	0.19	0.00	0.00
**188**	0.00	0.00	0.01	0.92	0.07	0.00	0.00	0.00
**189**	0.00	0.00	0.00	0.00	0.80	0.20	0.00	0.00
**190**	0.00	0.00	0.00	0.00	0.01	0.97	0.02	0.00
**191**	0.00	0.00	0.15	0.81	0.04	0.00	0.00	0.00
**192**	0.00	0.00	0.00	0.00	0.00	0.55	0.45	0.00
**193**	0.00	0.00	0.00	0.23	0.77	0.00	0.00	0.00
**194**	0.00	0.01	0.05	0.12	0.20	0.27	0.26	0.09
**195**	0.00	0.00	0.00	0.00	0.01	0.06	0.21	0.71
**196**	0.00	0.00	0.00	0.00	0.14	0.86	0.00	0.00
**197**	0.00	0.00	0.30	0.69	0.01	0.00	0.00	0.00
**198**	0.00	0.00	0.00	0.00	0.42	0.58	0.00	0.00
**199**	0.00	0.00	0.00	0.00	0.00	0.00	0.89	0.11
**200**	0.00	0.00	0.00	0.00	0.00	0.10	0.90	0.00
**201**	0.00	0.00	0.00	0.01	0.04	0.09	0.21	0.64
**202**	0.00	0.00	0.00	0.01	0.02	0.07	0.18	0.72
**203**	0.00	0.00	0.00	0.00	0.02	0.05	0.16	0.78
**204**	0.00	0.00	0.00	0.00	0.01	0.04	0.13	0.82
**205**	0.00	0.00	0.01	0.04	0.08	0.16	0.28	0.44
**206**	0.00	0.00	0.00	0.00	0.01	0.02	0.11	0.86
**207**	0.00	0.00	0.01	0.03	0.07	0.14	0.27	0.49
**208**	0.00	0.00	0.00	0.00	0.01	0.04	0.14	0.80
**209**	0.00	0.00	0.01	0.02	0.05	0.11	0.23	0.58
**210**	0.00	0.00	0.00	0.02	0.04	0.09	0.21	0.63
**211**	0.00	0.00	0.00	0.01	0.67	0.32	0.00	0.00
**212**	0.00	0.00	0.00	0.00	0.00	0.00	0.00	1.00
**213**	0.00	0.00	0.00	0.00	0.00	0.00	1.00	0.00
**214**	0.00	0.00	0.00	0.00	0.00	0.00	0.00	1.00
**215**	0.00	0.00	0.00	0.00	0.00	0.00	0.00	1.00
**216**	0.00	0.00	0.00	0.00	0.00	0.00	0.00	1.00
**217**	0.00	0.00	0.00	0.21	0.78	0.00	0.00	0.00
**218**	0.00	0.00	0.00	0.00	0.19	0.81	0.00	0.00
**219**	0.00	0.00	0.00	0.00	0.00	0.00	0.00	1.00
**220**	0.00	0.00	0.00	0.00	0.00	0.00	1.00	0.00
**221**	0.00	0.00	0.83	0.17	0.00	0.00	0.00	0.00
**222**	0.00	0.00	0.00	0.00	0.00	0.10	0.90	0.00
**223**	0.00	0.00	0.00	0.00	0.00	0.00	0.00	1.00
**224**	0.00	0.00	0.00	0.00	0.00	0.08	0.92	0.00
**225**	0.00	0.00	0.00	0.00	0.00	0.01	0.99	0.00
**226**	0.00	0.00	0.00	0.00	0.00	0.00	0.00	1.00
**227**	0.00	0.00	0.00	0.00	0.09	0.91	0.00	0.00
**228**	0.00	0.24	0.73	0.03	0.00	0.00	0.00	0.00
**229**	0.00	0.00	0.00	0.00	0.00	0.00	1.00	0.00
**230**	0.00	0.00	0.00	0.00	0.00	0.00	0.00	1.00
**231**	0.00	0.00	0.00	0.00	0.00	0.66	0.34	0.00
**232**	0.00	0.00	0.00	0.00	0.00	0.00	1.00	0.00
**233**	0.00	0.00	0.00	0.00	0.00	0.00	0.00	1.00
**234**	0.00	0.00	0.00	0.00	0.00	0.00	0.00	1.00
**235**	0.00	0.00	0.00	0.00	0.00	0.00	0.07	0.93
**236**	0.00	0.00	0.00	0.00	0.00	0.00	1.00	0.00
**237**	0.00	0.00	0.00	0.00	0.00	0.04	0.96	0.00
**238**	0.00	0.00	0.00	0.00	0.67	0.33	0.00	0.00
**239**	0.00	0.00	0.00	0.00	0.33	0.67	0.00	0.00
**240**	0.00	0.00	0.00	0.00	0.11	0.89	0.00	0.00
**241**	0.00	0.00	0.00	0.00	0.01	0.96	0.03	0.00
**242**	0.00	0.00	0.00	0.00	0.23	0.76	0.00	0.00
**243**	0.00	0.00	0.00	0.12	0.84	0.04	0.00	0.00
**244**	0.00	0.00	0.00	0.00	0.00	0.05	0.95	0.00
**245**	0.00	0.00	0.00	0.00	0.01	0.94	0.05	0.00
**246**	0.00	0.00	0.04	0.83	0.13	0.00	0.00	0.00
**247**	0.00	0.00	0.00	0.31	0.69	0.00	0.00	0.00
**248**	0.00	0.00	0.00	0.00	0.01	0.98	0.01	0.00
**249**	0.00	0.00	0.08	0.84	0.08	0.00	0.00	0.00
**250**	0.00	0.00	0.64	0.36	0.00	0.00	0.00	0.00
**251**	0.00	0.00	0.00	0.00	0.00	0.21	0.79	0.00
**252**	0.00	0.00	0.00	0.00	0.00	0.46	0.54	0.00
**253**	0.00	0.00	0.00	0.00	0.82	0.17	0.00	0.00
**254**	0.00	0.00	0.00	0.00	0.00	0.00	0.19	0.81
**255**	0.00	0.00	0.00	0.00	0.00	0.00	0.00	1.00
**256**	0.00	0.00	0.00	0.00	0.31	0.69	0.00	0.00
**257**	0.00	0.00	0.00	0.00	0.00	0.56	0.44	0.00
**258**	0.00	0.00	0.00	0.00	0.03	0.96	0.01	0.00
**259**	0.00	0.00	0.00	0.00	0.00	0.06	0.94	0.00
**260**	0.00	0.00	0.00	0.00	0.00	0.53	0.47	0.00
**261**	0.00	0.00	0.00	0.00	0.00	0.00	0.99	0.01
**262**	0.00	0.00	0.00	0.00	0.00	0.00	0.54	0.46
**263**	0.00	0.00	0.00	0.00	0.00	0.00	1.00	0.00
**264**	0.00	0.00	0.00	0.00	0.00	0.15	0.85	0.00
**265**	0.00	0.00	0.00	0.00	0.00	0.00	1.00	0.00
**266**	0.00	0.00	0.00	0.00	0.00	0.00	0.41	0.59
**267**	0.00	0.00	0.00	0.00	0.00	0.22	0.78	0.00
**268**	0.00	0.00	0.00	0.00	0.05	0.93	0.01	0.00
**269**	0.00	0.00	0.00	0.00	0.00	0.86	0.13	0.00
**270**	0.00	0.00	0.01	0.53	0.47	0.00	0.00	0.00
**271**	0.00	0.00	0.00	0.00	0.18	0.81	0.00	0.00
**272**	0.00	0.00	0.00	0.00	0.00	0.00	0.98	0.02
**273**	0.00	0.00	0.00	0.52	0.47	0.00	0.00	0.00
**274**	0.00	0.00	0.00	0.00	0.76	0.24	0.00	0.00
**275**	0.00	0.00	0.00	0.00	0.02	0.85	0.13	0.00
**276**	0.00	0.00	0.00	0.00	0.00	0.00	0.99	0.01
