# Supplementary material for: Protein Evolution by Molecular Tinkering: Diversification of the Nuclear Receptor Superfamily from a Ligand-Dependent Ancestor
Source: PLoS Biol. 2010 Oct 5;8(10):e1000497. doi: 10.1371/journal.pbio.1000497 (PMC2950128; doi:10.1371/journal.pbio.1000497)
Supplement: Table S4 — Protein structures and ligands used for calculating cavity volumes. (0.53 MB PDF) [file pbio.1000497.s013.pdf]

Table S4. Protein structures and ligands used for calculating cavity volumes

receptor	species	ligand	chain	pdb	resolution	volumes mean	volumes mode	volumes sd
AR	h	T	A	2Q7I	1.9	413	415	2
CAR	m	androstenol	A	1XNX	2.9	562	564	16
CAR	h	pregnanedione	B	1XV9	2.7	623	637	37
COUP-TF*	h	-	A	3CJW	1.5	173	203	34
DHR38	d	-	A	1PDU	2.3	28	30	4
ECR	Heliothis	ecdysone	D	2R40	2.4	674	671	4
ERα	h	E2	A	1ERE	3.1	450	453	11
ERβ	h	E2	A	2J7X	2.1	418	419	3
ERRα	h	NA	A	3D24	2.1	42	41	2
ERRα	h	antagonist CMPIMM	A	2PJ1	2.3	626	699	89
ERRγ	h	-	A	2ZBS	1.8	164	168	8
ERRγ	h	-	A	1KV6	2.7	253	264	27
ERRγ_with_TAM	h	4OHT	A	2P7Z	2.5	1022	906	220
FXR	r	Bile_acid	A	1OSV	2.5	1257	1321	360
GR	h	dex	A	1M2Z	2.5	538	542	14
hHNF4γ	h	palmitic_acid	A	1LV2	2.7	680	629	53
hLRH1	h	phosphatidylglycerol	A	1YOK	2.5	1633	1633	521
hSF1	h	phosphatidyl_choline	A	3F7D	2.2	2285	1980	726
LXR	h	oxysterol	A	1P8D	2.8	1018	906	187
mLRH1	m	-	A	1PK5	2.4	759	765	16
MR	h	DOC	A	2ABI	2.3	445	452	15
NGFIB	r	-	A	1YJE	2.4	37	38	2
Nurr1	h	-	A	1OVL	2.2	---	---	---
PPARδ	h	Fatty_acid	A	3GWX	2.4	1150	1205	298
PR	h	P4	A	1A28	1.8	489	493	11
PXR	h	-	A	1ILG	2.5	952	955	8
PXR	h	SR12813	A	1ILH	2.8	1107	1120	50
RARγ	h	Retinoic_acid	A	2LBD	2.1	488	492	14
Reverb-β**	h	heme	A	3CQV	1.9	606	579	61
RORα	h	chol._sulfate	A	1S0X	2.2	1969	1984	849
RORβ	R	Retinoic_acid	A	1N4H	2.1	888	798	309
TR	h	T3	X	1XZX	2.5	485	497	20
USP	heliothis	EPH	A	1G2N	1.7	2091	2104	463
USP	d	LPP	A	1HG4	2.4	1562	1346	495
VDR	h	VD	A	1DB1	1.8	800	808	26
Daf-12	nematode	Dafachronic_acid	A	3GYT	2.4	719	718	2
RXRα	h	atra	A	1FBY	2.3	574	585	36
ERRγ	h	BPA	A	2P7G	2.1	322	326	9
FTF1	d	-	A	2IZ2	2.8	191	232	50
CARβ	h	-	B	1XVP	2.6	608	620	25
CARβ	h	CID agonist	D	1XVP	2.6	646	649	3

* Modeled pocket with straightened H10 is larger (1)
** True pocket volume is larger but not detected because of open cavity mouth

1. Kruse S.W. et al., Identification of COUP-TFII Orphan Nuclear Receptor as a Retinoic Acid-Activated Receptor. PLoS Biol 6(9): e227 (2008)

VOIDOO software in probe-occupied mode was used to calculate cavity volumes, with the centroid of the bound ligand or a manually defined point as a starting locus for cavity searches. Mean and mode cavity volumes with standard deviation are listed for cavity calculations using ten random orientations of the protein with ten different “van der Waals growth factors” ranging from 1.1-1.3.
