# Supplementary material for: Protein Evolution by Molecular Tinkering: Diversification of the Nuclear Receptor Superfamily from a Ligand-Dependent Ancestor
Source: PLoS Biol. 2010 Oct 5;8(10):e1000497. doi: 10.1371/journal.pbio.1000497 (PMC2950128; doi:10.1371/journal.pbio.1000497)
Supplement: Table S5 — Cavity volumes of modeled nuclear receptors using different templates (0.75 MB PDF) [file pbio.1000497.s014.pdf]

Table S5. Cavity volumes of modeled nuclear receptors.

We created homology models of the LBDs of AqNR1 (gi: 167859601, residues 404-534), annelid ER (gi:186908731, residues 231-479), Branchistoma SR (gi: 170178459, residues 298-532) and Branchiostoma ER (gi: 170178461, residues 250-504). To estimate the effect of template choice on our results, we used several LBD structural templates for each model (see below). Human ERα , ERR3, and ERRα were used as templates for all models. HNF4α was used as template only for AqNR1. We used the global alignment described for the phylogenetic analysis as the alignment for the structural models. Alignment uncertainty was limited to the termini, neither of which border the ligand binding pocket.

Table 1: Templates used for homology models

LBD	ligand	pdb:chain	pocket volume (Å^3)	% pairwise identity AqNR1	annER	BraSR	BraER
ERα	estrogen	1ERE:A	447	30.2	30.2	32.5	31.3
ERR3	apo	1KV6:A	262	27.9	27.9	27.4	31
ERRα	apo	3D24:A	42	28.8	28.8	27.8	26.7
HNF4α	DAO	1MV7:A	680	32.2	22.3	25.6	22.4

We generated ten models for every protein with Modeller 9.7 using the default parameters[1]. We then visually inspected the models for artifacts (e.g. "knotting") and removed any unrealistic models. The models were further assessed using RamPage[2] as distributed with CCP4i[3]. Only models with >95% of residues in the preferred region and <1% in the outlier region of the Ramachandran map were accepted. No models generated from the ERRα template passed these criteria for AqNR1, annER or braSR. We calculated the pocket volumes of the remaining models using the standard VOIDOO protocol.

Table 2: Dependence of calculated pocket volume on template pocket volume.

		AqNR1		annER		BraER		BraSR	
template	template pocket	mean	sd	mean	sd	mean	sd	mean	sd
ERRa	42	*	*	*	*	365	158	*	*
ERR3	262	604	228	419	113	565	203	467	201
ERα	447	641	300	574	72	701	440	438	43
HNF4α	680	485	109	-	-	-	-	-	-

* Template did not yield models that passed quality standards.
\- No homology model generated with this template.

While the magnitudes of the cavity volumes were sensitive to the choice of template, in no case was the cavity entirely closed. The average volume for all models was always >350 Å^3. To verify that this was not because Modeller systematically over-estimates cavity volumes, we also built models of ERRα (known volume = 42 Å^3) using human ERα as a template. If Modeller led to systematically larger cavity volumes, the calculated volumes of the models would be > 42 Å^3. This was not observed. In fact, Modeller underestimated the ERRα cavity volume, for none of the ERRα models had defined cavities at the ligand binding site.

All receptors in this analysis were modeled with no ligand in the pocket. The estimated cavity volume for AqNR1 in this analysis differs from that observed in the homology we generated using Insight II software, because the latter model was generated with palmitic acid (the FA most abundantly bound by AqNR1 in our experiments) docked in the pocket.

References:

1.Eswar, N. et al. Comparative protein structure modeling using MODELLER. *CurrProtoc Protein Sci* **Chapter 2**, Unit 2.9 (2007).
2.Lovell, S.C. et al. Structure validation by Calpha geometry: phi, psi and Cbeta deviation. *Proteins: Structure, Function, and Genetics* **50**, 437-450 (2003).
3.Winn, M.D. An overview of the CCP4 project in protein crystallography: an example of a collaborative project. *J Synchrotron Rad* **10**, 23-25 (2002).
