# Supplementary material for: Protein Evolution by Molecular Tinkering: Diversification of the Nuclear Receptor Superfamily from a Ligand-Dependent Ancestor
Source: PLoS Biol. 2010 Oct 5;8(10):e1000497. doi: 10.1371/journal.pbio.1000497 (PMC2950128; doi:10.1371/journal.pbio.1000497)
Supplement: Table S6 — Key to nuclear receptor nomenclature. (0.44 MB PDF) [file pbio.1000497.s015.pdf]

Table S6. Nuclear receptor nomenclature.

Receptor Abbreviation	Receptor	Nomenclature
DAX-1	Dosage-sensitive sex reversal, adrenal hypoplasia critical region, on chromosome X, gene 1	NR0B1
SHP	Small heterodimer partner	NR0B2
ThRα	Thyroid hormone receptor-α	NR1A1
ThRβ	Thyroid hormone receptor-β	NR1A2
RARα	Retinoic acid receptor-α	NR1B1
RARβ	Retinoic acid receptor-β	NR1B2
RARγ	Retinoic acid receptor-γ	NR1B3
PPARα	Peroxisome proliferator-activated receptor- α	NR1C1
PAPRβ	Peroxisome proliferator-activated receptor- β	NR1C2
PPARγ	Peroxisome proliferator-activated receptor- γ	NR1C3
Rev-ErbAα	Rev-ErbAα	NR1D1
Rev-ErbAβ	Rev-ErbAβ	NR1D2
E75	Ecdysone-inducible protein E75	NR1D3
E78	Ecdysone-inducible protein E78	NR1E1
RORα	RAR-related orphan receptor-α	NR1F1
RORβ	RAR-related orphan receptor-β	NR1F2
RORγ	RAR-related orphan receptor-γ	NR1F3
HR3	Hormone receptor 3	NR1F4
EcR	Ecdysone Receptor	NR1H1
LXRβ	Liver X receptor-β	NR1H2
LXRα	Liver X receptor-α	NR1H3
VDR	Vitamin D receptor	NR1I1
PXR	Pregnane X receptor	NR1I2
CAR	Constitutive androstane receptor	NR1I3
HR96	Hormone receptor-like 96	NR1J1
FXR	Farnesoid X receptor	NR1H4
HNF4α	Hepatocyte nuclear factor-4-α	NR2A1
RXRα	Retinoid X receptor-α	NR2B1
RXRβ	Retinoid X receptor-β	NR2B2
RXRγ	Retinoid X receptor-γ	NR2B3
usp	Ultraspiracle	NR2B4
TR2	Testicular receptor 2	NR2C1
TR4	Testicular receptor 4	NR2C2
HR78	Hormone receptor-like 78	NR2D1
TLL	Tailless	NR2E1
TLX	Human homologue of the Drosophila tailless gene	NR2E1
PNR	Photoreceptor cell-specific nuclear receptor	NR2E3
dsf	Dissatisfaction	NR2E4
FAX1	Defective fasciculation of axons-1	NR2E5
COUP-TFI	Chicken ovalbumin upstream promoter-transcription factor	NR2F1
COUP-TFII	Chicken ovalbumin upstream promoter-transcription factor	NR2F2
svp	Seven-up	NR2F3
EAR-2	V-erbA-related gene|V-erbA-related	NR2F6
ERα	Estrogen receptor-α	NR3A1
ERβ	Estrogen receptor-β	NR3A2
ERRα	Estrogen-related receptor-α	NR3B1
ERRβ	Estrogen-related receptor-β	NR3B2
ERRγ	Estrogen-related receptor-γ	NR3B3
GR	Glucocorticoid receptor	NR3C1
MR	Mineralocorticoid receptor	NR3C2
AR	Androgen Receptor	NR3C4
PR	Progesterone receptor	NR3C3
NGFIB	Nerve Growth factor IB	NR4A1
NURR	Nuclear receptor related 1	NR4A2
NOR1	Neuron-derived orphan receptor 1	NR4A3
HR38	Hormone receptor-like 38	NR4A4
SF1	Steroidogenic receptor 1	NR5A1
LRH-1	Liver Receptor Homolog 1	NR5A2
FTZ-F1	Fushi tarazu F1-like	NR5A3
GCNF	Germ Cell Nuclear Factor	NR6A1
GRF	(GCNF)-related factor	NR6A1

Nuclear Receptors Nomenclature Committee. A Unified Nomenclature System for the Nuclear Receptor Superfamily. *Cell* **97**, 161-163 (1999).
