# Supplementary material for: Protein Evolution by Molecular Tinkering: Diversification of the Nuclear Receptor Superfamily from a Ligand-Dependent Ancestor
Source: PLoS Biol. 2010 Oct 5;8(10):e1000497. doi: 10.1371/journal.pbio.1000497 (PMC2950128; doi:10.1371/journal.pbio.1000497)
Supplement: Table S7 — Protein data bank accession numbers for structures referred to in the text. (0.30 MB PDF) [file pbio.1000497.s016.pdf]

Table S7. Protein data bank accession numbers for structures

Structures of receptors with ligands

Receptor	Ligand	PDB IDs
human HNF4α	myristic acid	3FS1
mouse RXRα	retinoic acid	1XDKa
mouse RARβ	retinoic acid	1XDKb
human ERα	estradiol	1GWR
human PPARα	3-{5-methoxy-1-[(4-methoxyphenyl)sulfony l]- 1H-indol-3-yl}propanoic acid	3ET1

Structures of constitutively active receptors

Receptor	PDB Ids
rat NGFIB	1YJE
human ERRα	3G24
FTZF1	2IZ2
cgERR	TBD
